# Skeletal muscle overuse injury: pathophysiological mechanisms, molecular pathways, and rehabilitation strategies

**DOI:** 10.3389/fphys.2026.1832044

**Published:** 2026-06-02

**Authors:** Guoliang Wu, Na Li

**Affiliations:** Guangxi Science and Technology Normal University, Laibin, China

**Keywords:** evidence hierarchy, extracellular matrix remodeling, inflammation, load management, mitochondrial dysfunction, oxidative stress, rehabilitation, satellite cells

## Abstract

Skeletal muscle overuse injury (OUI) is a load-related condition that develops when repeated mechanical loading exceeds the adaptive and reparative capacity of skeletal muscle. Unlike acute traumatic injury or delayed-onset muscle soreness after unaccustomed eccentric exercise, chronic skeletal muscle OUI is characterized by recurrent subthreshold loading, insufficient recovery, persistent low-grade inflammation, impaired regeneration, and maladaptive remodeling. This narrative review summarizes and critically appraises current evidence on the conceptual boundaries, pathophysiological mechanisms, molecular pathways, and rehabilitation strategies of skeletal muscle OUI. Particular emphasis is placed on distinguishing direct skeletal muscle evidence from indirect or extrapolative evidence derived from acute injury models, adjacent musculoskeletal disorders, disease models, or preclinical studies. Key mechanisms include myofiber microdamage, satellite-cell-mediated repair, extracellular matrix remodeling, inflammatory signaling, oxidative stress, mitochondrial dysfunction, protein turnover, and myogenic transcriptional regulation. Current management remains centered on individualized load modification, graded rehabilitation, correction of biomechanical contributors, and criteria-based return to activity. Pharmacological and physical modalities may provide adjunctive symptom control in selected cases, whereas regenerative, gene-based, wearable-sensor-based, and artificial-intelligence-assisted approaches remain emerging or experimental for chronic skeletal muscle OUI. By integrating mechanistic evidence with rehabilitation practice and evidence appraisal, this review provides a focused framework for understanding, preventing, and managing skeletal muscle OUI.

## 1 Introduction

Skeletal muscle is a highly adaptable tissue that supports movement, posture, force generation, metabolic regulation, thermoregulation, and glucose homeostasis (Wendt et al., 2007). Its contractile function depends on coordinated excitation–contraction coupling and Ca²^+^ cycling, and disruption of this regulation can impair force production and relaxation (Stammers et al., 2015). Under physiological loading and adequate recovery, skeletal muscle undergoes adaptive remodeling through satellite-cell-mediated myofiber repair, hypertrophy, extracellular matrix (ECM) remodeling, and metabolic adaptation (Peck et al., 2022). These responses are influenced by training status, as trained and untrained individuals may show different repair-related biomarker responses after exercise (Scarpelli et al., 2024). Endurance training further supports fatigue resistance and metabolic efficiency by enhancing mitochondrial enzyme activity (Gollnick and Saltin, 1982; Henriksson, 1992; Spina et al., 1996). However, when repetitive mechanical loading exceeds the adaptive and reparative capacity of skeletal muscle, particularly under conditions of high strain or insufficient recovery, the balance may shift from adaptive remodeling toward skeletal muscle overuse injury (OUI) (Armstrong et al., 1991).

Skeletal muscle OUI is a load-related condition that should be distinguished from acute traumatic injury, delayed-onset muscle soreness after unaccustomed eccentric exercise, tendinopathy, and broader musculoskeletal overuse disorders. A defining feature is the gradual accumulation of microdamage and progressive impairment of repair capacity, rather than the abrupt tissue disruption typical of acute trauma (Aicale et al., 2018). In this review, skeletal muscle OUI refers primarily to recurrent myofiber microdamage, persistent soreness or fatigue, impaired contractile function, incomplete regeneration, maladaptive inflammation, and remodeling of the muscle ECM. Tendon and cartilage changes may coexist clinically, but they are considered adjacent musculoskeletal conditions rather than core skeletal muscle mechanisms (Khan et al., 1999). If not adequately managed, skeletal muscle OUI may contribute to persistent pain, functional limitation, reduced muscle strength, increased fall risk in older adults, and impaired training continuity, force production, movement efficiency, and sport-specific performance in athletes (Smith, 1996; Weerapong et al., 2005). Some athletes may also experience psychological distress or identity disruption during prolonged rehabilitation or forced retirement (Smith, 1996).

Although the clinical relevance of skeletal muscle OUI is increasingly recognized, its mechanisms remain incompletely defined. A major problem is that chronic skeletal muscle OUI is often interpreted using evidence from acute eccentric injury, delayed-onset muscle soreness, tendinopathy, cartilage disorders, or other musculoskeletal conditions. These models are informative but not directly equivalent to chronic load-related skeletal muscle pathology. Important knowledge gaps remain regarding the distinction between acute and chronic injury, the regulation of molecular pathways such as NF-κB, p38 MAPK, MyoD, and MyoG, and the translation of emerging rehabilitation approaches, including stem cell therapies and real-time monitoring with wearable devices. These gaps limit the precision of prevention, rehabilitation, and clinical management.

With advances in molecular biology, muscle regeneration research, and rehabilitation science, studies of skeletal muscle OUI have increasingly shifted from macroscopic tissue injury toward cellular and molecular mechanisms. Key areas include satellite cell self-renewal and differentiation (Dumont et al., 2015), fibrosis associated with dysregulated ECM remodeling (Fang et al., 2023), and oxidative-stress-related mitochondrial impairment (Powers and Jackson, 2008). Current rehabilitation remains centered on load management, graded exercise, and physical therapy, whereas gene-based interventions and intelligent monitoring technologies should be regarded as emerging or experimental approaches that require further validation in skeletal muscle OUI (Nadler et al., 2004; Stilhano et al., 2015; Morelli et al., 2018; Chen et al., 2025). In this context, reviewing the fundamental concepts, pathophysiological mechanisms, molecular pathways, and rehabilitation strategies of skeletal muscle OUI may help clarify current evidence, identify translational limitations, and support more precise prevention and rehabilitation strategies.

This review focuses primarily on skeletal muscle OUI. Tendon, cartilage, ligament, and peripheral nerve abnormalities are discussed only as adjacent musculoskeletal contexts or secondary consequences of altered loading, not as interchangeable models of skeletal muscle pathology (Bylak and Hutchinson, 1998; Khan et al., 1999; Aicale et al., 2018; Moo et al., 2022). Because evidence for chronic skeletal muscle OUI is heterogeneous, this review distinguishes direct skeletal muscle evidence from indirect or extrapolative evidence derived from acute injury models, tendon or cartilage disorders, volumetric muscle loss, burn injury, genetic myopathies, or other adjacent fields (Cheung et al., 2003; Powers and Jackson, 2008; Tidball and Villalta, 2010; Dumont et al., 2015; Aicale et al., 2018; Wohlgemuth et al., 2023). To make this distinction explicit, **Table 1** summarizes the directness, evidence source, and limitations of the main mechanisms and interventions discussed in this review.

**Table 1:** Evidence directness and limitations of key mechanisms and approaches discussed in skeletal muscle overuse injury (OUI) topic.

Types	Main evidence source	Relevance to skeletal muscle OUI	Main limitation	References
Acute eccentric microdamage and DOMS	Human and animal acute exercise-induced muscle damage models	Indirect/acute-model evidence	Mechanistically relevant to myofiber disruption and inflammation but does not fully reproduce chronic skeletal muscle OUI	(Armstrong et al., 1991; Cheung et al., 2003; Tidball, 2011; Peake et al., 2017)
Recurrent overload and training-load imbalance	Sports medicine, workload monitoring, overtraining, and mechanical fatigue literature	Indirect clinical-workload evidence	Often evaluates injury risk, soreness, or workload associations rather than tissue-level skeletal muscle pathology	(Kibler et al., 1992; Bahr and Krosshaug, 2005; Meeusen et al., 2013; Halson, 2014; Drew and Finch, 2016; Soligard et al., 2016; Aicale et al., 2018; Edwards, 2018; Brenner et al., 2024)
Satellite cell activation and myogenic repair	Skeletal muscle regeneration, loading adaptation, and repair models	Indirect mechanistic evidence	Mechanistically strong for muscle repair, but chronic OUI-specific human evidence remains limited	(Hill et al., 2003; Tidball and Villalta, 2010; Tidball, 2011; Yablonka-Reuveni, 2011; Chang and Rudnicki, 2014; Motohashi and Asakura, 2014; Dumont et al., 2015; Murach et al., 2021; Kong et al., 2024)
ECM remodeling and fibrosis	Skeletal muscle ECM biology, muscle injury, burn injury, volumetric muscle loss, rotator cuff, and OA-associated muscle models	Extrapolative evidence	Supports a plausible remodeling mechanism, but not all models are caused by repetitive overuse loading	(Kjaer, 2004; Gumucio et al., 2013; Gigliotti et al., 2017; Brightwell et al., 2020; Corona et al., 2020; Fang et al., 2023; Wohlgemuth et al., 2023; Yu et al., 2025)
Inflammation and oxidative stress	Exercise-induced muscle damage, muscle injury, inflammatory disease, metabolic stress, and cell models	Indirect mechanistic evidence	Inflammation and ROS can be adaptive or maladaptive depending on timing, magnitude, and tissue context; causal hierarchy in chronic OUI remains incompletely defined	(Tidball, 2005; Powers and Jackson, 2008; Khansari et al., 2009; Ambrosio et al., 2014; Jaiswal et al., 2015; Liu et al., 2017; Molinari et al., 2017; Peake et al., 2017; Choi, 2018; Castanheira and Kubes, 2019; Wang et al., 2021; Torres-Ruiz et al., 2023; Tu and Li, 2023; Bonato et al., 2024)
Pharmacological interventions	Acute skeletal muscle injury, soft-tissue healing, tendinopathy, and adjacent musculoskeletal evidence	Adjunctive/indirect evidence	May support short-term symptom control in selected cases, but disease-modifying effects in chronic skeletal muscle OUI are not established	(Garrick, 2011; Lisowska et al., 2018; Morelli et al., 2018; Solaiman et al., 2024)
Physical therapy modalities	Clinical rehabilitation practice, pain modulation literature, ultrasound studies, and adjacent musculoskeletal evidence	Adjunctive/indirect evidence	Effects are parameter-dependent and often supported by heterogeneous or indirect evidence rather than direct skeletal muscle OUI trials	(Nadler et al., 2004; Weerapong et al., 2005; Wong et al., 2007; Uddin et al., 2021)
Regenerative and gene-based therapies	Preclinical muscle regeneration, satellite cell biology, growth factor, angiogenesis, and muscle disease models	Experimental evidence	Biologically promising, but clinical safety, delivery, specificity, durability, and direct efficacy in skeletal muscle OUI remain unresolved	(Hill et al., 2003; Messina et al., 2007; Wong et al., 2007; Yablonka-Reuveni, 2011; Chang and Rudnicki, 2014; Motohashi and Asakura, 2014; Shimizu-Motohashi and Asakura, 2014; Dumont et al., 2015; Stilhano et al., 2015; Lisowska et al., 2018; Farooq et al., 2021; San Emeterio et al., 2021; Uddin et al., 2021; Kong et al., 2024; Solaiman et al., 2024; Wei et al., 2024)
Wearable sensors and AI-assisted rehabilitation	Technology validation, fatigue monitoring, workload tracking, chronic pain AI, and sports monitoring studies	Emerging evidence	Useful for monitoring and decision support, but clinical outcome validation in skeletal muscle OUI remains limited	(Benson et al., 2020; Truppa et al., 2020; Rebelo et al., 2023; Khan et al., 2024; Chen et al., 2025; Di Paco and Cannataro, 2025; Rubio-Zarapuz et al., 2025)

Evidence relevance was categorized as indirect, extrapolative, adjunctive/indirect, experimental, or emerging according to whether the supporting evidence was derived from acute skeletal muscle injury models, chronic load-monitoring studies, adjacent musculoskeletal disorders, preclinical studies, or technology validation research. Direct clinical evidence specific to chronic skeletal muscle OUI remains limited.

This review is organized into three parts. First, it defines skeletal muscle OUI and clarifies its conceptual boundaries. Second, it summarizes pathophysiological and molecular mechanisms, including myofiber injury, satellite-cell-mediated repair, ECM remodeling, inflammation, oxidative stress, protein turnover, and stress-responsive myogenic transcriptional regulation. Third, it evaluates rehabilitation and emerging therapeutic strategies according to evidence strength, clinical readiness, and relevance to skeletal muscle OUI.

## 2 Literature search strategy and scope

This article was designed as a narrative review rather than a systematic review. The objective was to summarize and critically appraise representative, mechanistically informative, and clinically relevant evidence on the concepts, pathophysiological mechanisms, molecular pathways, and rehabilitation strategies relevant to skeletal muscle OUI. Database searches covered publications from database inception to April 2026.

Key literature was identified by searching PubMed, Web of Science, and Scopus, together with manual screening of reference lists from relevant reviews and original studies. The search terms included combinations of “skeletal muscle,” “myofiber,” “muscle injury,” “overuse injury,” “exercise-induced muscle damage,” “repetitive loading,” “overtraining,” “inflammation,” “oxidative stress,” “satellite cells,” “extracellular matrix,” “fibrosis,” “rehabilitation,” “wearable sensors,” and “artificial intelligence.”.

Peer-reviewed English-language articles were prioritized. Preprints, conference abstracts, non-peer-reviewed sources, and non-English publications were not used as primary evidence unless they provided essential contextual information. Articles were considered if they addressed skeletal muscle OUI, recurrent loading, muscle repair, inflammation, oxidative stress, mitochondrial dysfunction, ECM remodeling, rehabilitation, or emerging monitoring and therapeutic technologies.

Because this was a narrative review, no formal risk-of-bias assessment, PRISMA flow diagram, or quantitative synthesis was performed. When conflicting evidence was identified, greater interpretive weight was given to direct skeletal muscle evidence, human or clinically relevant studies, mechanistic consistency, and relevance to chronic load-related injury rather than acute injury alone. Evidence from adjacent or indirect models was included only when it clarified general mechanisms and is interpreted cautiously throughout the review. **Table 1** summarizes the evidence directness, main evidence sources, and limitations of the mechanisms and approaches discussed in this review.

## 3 Findings

### 3.1 Fundamental concepts of skeletal muscle OUI

#### 3.1.1 Skeletal muscle adaptation and transition to overuse injury

Skeletal muscle adaptation depends on coordinated remodeling of myofibers, extracellular matrix, metabolic capacity, vascular support, and neuromuscular control (Henriksson, 1992; Wendt et al., 2007; Schoenfeld, 2010; Stammers et al., 2015; Murach et al., 2021; Peck et al., 2022; Scarpelli et al., 2024). Under appropriate loading and recovery conditions, mechanical stimuli can promote satellite cell activation, protein turnover, ECM remodeling, and metabolic adaptation, thereby improving force production and tissue tolerance (Schoenfeld, 2010; Murach et al., 2021; Peck et al., 2022; Scarpelli et al., 2024). However, these adaptive responses have physiological limits. When repeated loading is excessive, progresses too rapidly, or occurs with insufficient recovery, adaptive remodeling may shift toward recurrent myofiber microdamage, persistent inflammatory signaling, impaired regeneration, and maladaptive ECM remodeling (Armstrong et al., 1991; Kibler et al., 1992; Tidball, 2011; Meeusen et al., 2013; Peake et al., 2017; Aicale et al., 2018; Edwards, 2018). Fatigue-related reductions in active muscle stiffness and force-generating capacity may further reduce tolerance to repeated stretch or shear forces, increasing vulnerability to recurrent injury (Murgia, 2013; Edwards, 2018; Evangelidis et al., 2023). This transition from adaptive remodeling to failed repair provides the conceptual basis for chronic skeletal muscle OUI.

#### 3.1.2 Definition and classification of overuse injury

Skeletal muscle OUI can be defined as a load-related pathological state in which repeated or excessive mechanical stress produces cumulative myofiber microdamage that exceeds the reparative capacity of skeletal muscle (Aicale et al., 2018). It differs from acute traumatic injury, which usually results from a discrete event such as direct impact, sudden overstretching, or forceful contraction. OUI typically develops gradually, often after increases in load intensity, frequency, duration, or insufficient recovery. In this review, OUI refers specifically to skeletal muscle pathology, including recurrent myofiber microdamage, persistent soreness or fatigue, impaired force production, incomplete regeneration, and maladaptive remodeling (Aicale et al., 2018).

In skeletal muscle, OUI commonly manifests as recurrent microdamage, chronic fatigue, soreness, impaired contractile function, and reduced tolerance to repeated loading. Evidence suggests that muscle fatigue is an important contributor to such injuries—the biomechanical properties (e.g., active stiffness) of fatigued muscles decrease significantly, decreasing their resistance to stretching and shearing forces, thereby increasing injury risk. Eccentric contractions are particularly relevant to experimental models of muscle damage because lengthening under load imposes high mechanical strain on myofibers (Edwards, 2018; Evangelidis et al., 2023). Furthermore, excessive overtraining leads to tissue fatigue accumulation, which further hinders the muscle’s ability to repair, creating a harmful cycle of “injury–fatigue–re-injury.” Gradual load progression and sufficient recovery may help interrupt this cycle and reduce recurrence risk (Kibler et al., 1992; Murgia, 2013).

Tendon, cartilage, ligament, and peripheral nerve disorders may coexist with skeletal muscle OUI in clinical practice, but they are discussed here only as adjacent or secondary contexts because their tissue biology and repair mechanisms differ from those of skeletal muscle (Bylak and Hutchinson, 1998; Khan et al., 1999; Aicale et al., 2018; Moo et al., 2022). Accordingly, classification in this review is based on skeletal muscle-centered features, including recurrent myofiber microdamage, persistent fatigue or soreness, impaired force generation, incomplete regeneration, maladaptive inflammation, and ECM remodeling.

Evidence suggests that OUI in athletes is often associated with inappropriate load progression, incomplete recovery, faulty movement patterns, and premature return to activity after a previous injury. This highlights the importance of early load modification, adequate recovery, and targeted rehabilitation to reduce the risk of chronic maladaptation (el-Khoury et al., 1997). Etiologically, skeletal muscle OUI is usually multifactorial and may result from the combined effects of inappropriate training progression, faulty movement patterns, excessive training volume or intensity, and inadequate recovery (Meeusen et al., 2013). These factors together disrupt the physiological load–repair balance and may promote the development of OUI.

#### 3.1.3 Clinical characteristics and functional impact of skeletal muscle overuse injury

##### 3.1.3.1 Key clinical manifestations

Skeletal muscle OUI is characterized by local muscle pain, soreness, stiffness, fatigue, reduced force production, and impaired tolerance to repeated activity. Pain or soreness is commonly localized to repeatedly loaded muscle groups and may worsen with continued activity while improving with rest (Arnold and Moody, 2018). Pain may increase when repeated contractions load the affected muscle during movement. Symptoms may improve during rest as mechanical loading decreases. Nevertheless, as the injury worsens, pain progressively becomes persistent and may progress to a dull ache at rest, suggesting that tissue repair capacity has been exceeded (Weerapong et al., 2005). Repetitive microtrauma may also produce local edema and non-infectious inflammatory signs, such as mild warmth or redness. This may result from recurrent loading-induced microtears, activation of local inflammatory pathways, and cytokine release, which together contribute to edema and reduced tissue elasticity (Noonan et al., 1999). At the same time, patients may show reduced activity tolerance and develop fatigue during routine tasks, such as climbing stairs or squatting, thus needing frequent rest breaks. In more severe cases, even low-intensity activity may become difficult to sustain (Noonan et al., 1999). Skeletal muscle OUI may reduce flexibility, range of motion, and movement efficiency, particularly when pain, stiffness, or protective neuromuscular inhibition alters normal movement patterns (Behm et al., 2016). Impaired contractile function may also contribute to reduced muscle strength. Reduced strength and impaired muscle activation may occur in some patients, although the magnitude of functional loss varies according to injury severity, muscle group, training history, and timing of assessment (Noonan et al., 1999). Delayed-onset muscle soreness (DOMS) may accompany acute eccentric-exercise-induced muscle damage, but DOMS should not be equated with chronic skeletal muscle OUI (Cheung et al., 2003). In this review, DOMS is used only as an acute symptom model for exercise-induced muscle damage, whereas chronic skeletal muscle OUI is defined by recurrent loading, incomplete recovery, and maladaptive remodeling.

##### 3.1.3.2 Multidimensional impact

The impact of skeletal muscle OUI extends beyond the affected muscle and may influence daily activity, sport performance, and psychological well-being, particularly in athletes.

In the general population, mild skeletal muscle OUI may initially cause only exercise-related discomfort, but inadequate management can contribute to persistent pain, recurrent activity limitation, reduced training tolerance, and compensatory movement patterns. In older adults, decreased muscle strength can increase the likelihood of falls. In athletes, pain, reduced force production, impaired coordination, and disrupted training continuity may compromise sport-specific performance (Weerapong et al., 2005). Rehabilitation duration varies according to injury severity, recurrence risk, sport demands, and load management strategy and should not be presented as a fixed timeline (Hägglund et al., 2005).

Prolonged symptoms, restricted participation, and uncertainty during rehabilitation may negatively affect psychological well-being, particularly in athletes. Evidence suggests that several athletes with OUIs face symptoms of anxiety and depression, defined by worry about rehabilitation outcomes, low mood, and poor sleep quality. Some may also experience reduced self-worth when they are unable to participate in sport (Smith, 1996). In professional athletes, prolonged inability to compete or forced retirement may contribute to identity disruption. Athletes who strongly identify with their sporting role may experience loss, uncertainty, or reduced self-esteem when they are removed from competition (Furie et al., 2023). Psychological support from clinicians, coaches, and athletic trainers may help reduce distress during rehabilitation, although the magnitude of benefit likely depends on injury type, athlete population, and study design (Yang et al., 2014). These observations support the inclusion of psychosocial considerations in rehabilitation planning, without implying that social support alone determines recovery outcomes (Yang et al., 2014).

### 3.2 Pathophysiological mechanisms of skeletal muscle OUI

Under physiological loading conditions, skeletal muscle has a substantial capacity for repair and adaptation. However, OUI occurs when repetitive or excessive loading exceeds the reparative capacity of skeletal muscle (Armstrong et al., 1991; Kibler et al., 1992). OUI progression involves myofiber disruption, altered cellular responses, and activation of molecular pathways that may lead to structural and functional impairment (Kjaer, 2004). Understanding these mechanisms, particularly the distinction between acute eccentric injury and chronic skeletal muscle OUI, is important for developing more precise prevention and rehabilitation strategies.

#### 3.2.1 Acute and chronic mechanisms of overuse injury

**Figure 1:**
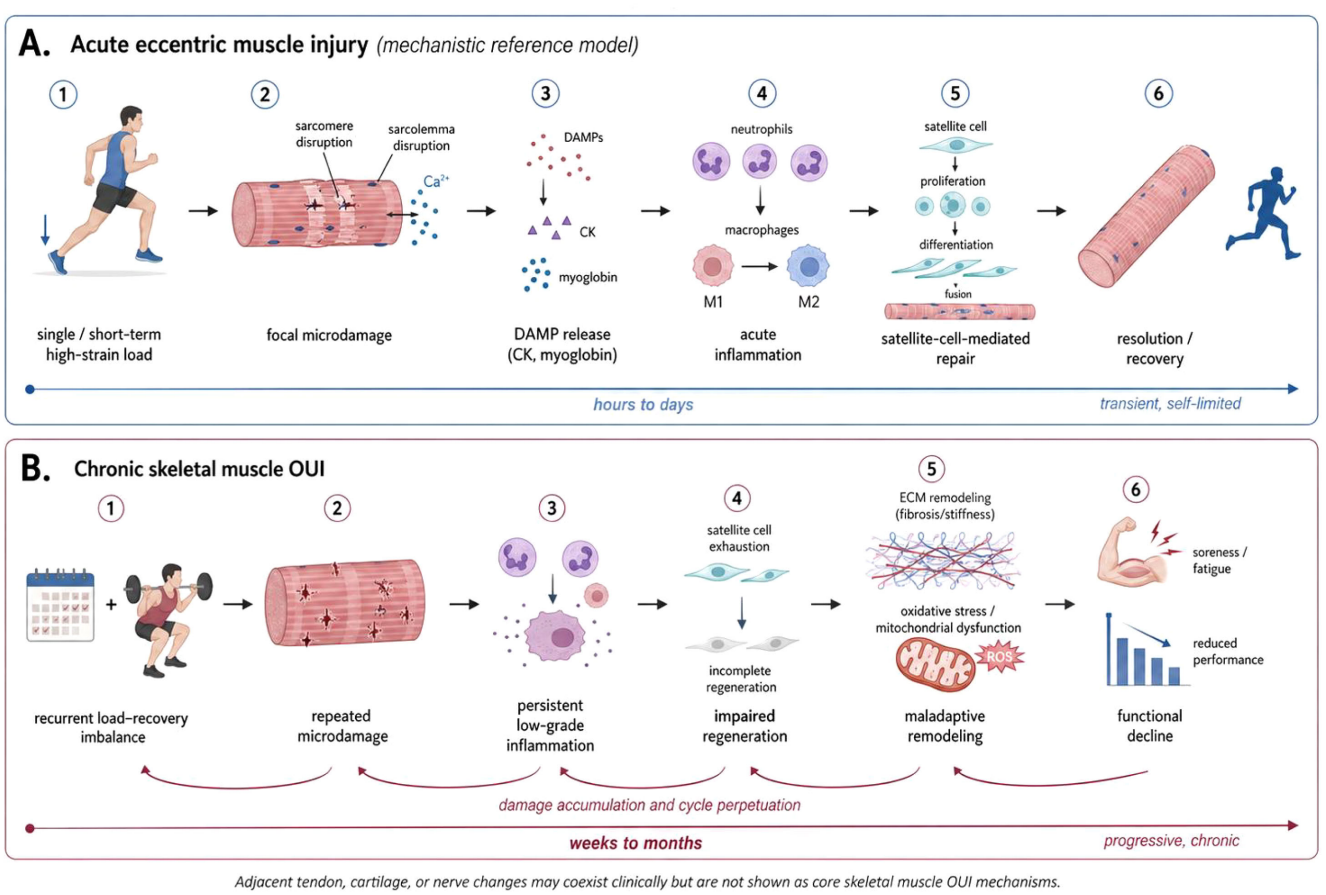
Acute eccentric muscle injury and chronic skeletal muscle OUI. **(A)** Acute eccentric injury is presented as a mechanistic reference model involving short-term myofiber microdamage, DAMP release, inflammatory cell recruitment, macrophage phenotype transition, and satellite-cell-mediated repair. **(B)** Chronic skeletal muscle OUI reflects recurrent loading, insufficient recovery, persistent low-grade inflammation, impaired regeneration, and maladaptive ECM remodeling. Adjacent tendon, cartilage, or nerve changes may coexist clinically but are not shown as core skeletal muscle OUI mechanisms.

Acute eccentric-exercise-induced muscle injury, DOMS, recurrent subthreshold overload, overtraining-related maladaptation, and chronic skeletal muscle OUI should be treated as related but distinct concepts (Kibler et al., 1992; Cheung et al., 2003; Aicale et al., 2018). Acute eccentric injury and DOMS are useful experimental models for studying early myofiber microdamage and inflammatory repair, but they do not fully reproduce chronic skeletal muscle OUI (Cheung et al., 2003; Tidball, 2011; Peake et al., 2017). Chronic skeletal muscle OUI more often reflects repeated subthreshold loading, insufficient recovery, maladaptive inflammation, impaired satellite cell function, extracellular matrix dysregulation, and progressive loss of tissue quality (Aicale et al., 2018; San Emeterio et al., 2021; Wohlgemuth et al., 2023). Overuse injuries arise from a complex interplay between mechanical loading and biological repair and can be broadly classified into acute and chronic forms based on the temporal pattern of stress and the body’s adaptive capacity. While both types share an initial imbalance between load and tissue tolerance, their underlying mechanisms, inflammatory characteristics, and clinical outcomes differ fundamentally. The following section uses acute eccentric-exercise-induced muscle injury as a mechanistic reference model for early myofiber microdamage, inflammation, and repair, rather than as a direct equivalent of chronic skeletal muscle OUI. Chronic overuse injury, characterized by cumulative microdamage and persistent low-grade inflammation, is addressed in **Figure 1**.

##### 3.2.1.1 Acute injury

Acute eccentric-exercise-induced muscle damage is discussed here as a mechanistic reference model rather than as a direct equivalent of chronic skeletal muscle OUI. It is useful for understanding sarcolemmal disruption, Ca^2+^ disturbance, inflammatory cell recruitment, and early regenerative signaling after high-strain loading. However, chronic skeletal muscle OUI more commonly develops through repeated subthreshold overload, insufficient recovery, and maladaptive remodeling rather than through a single acute eccentric insult. In this model, high-strain eccentric loading can induce myofiber microdamage, acute inflammatory cell recruitment, and early activation of repair pathways in a time-dependent sequence (Cheung et al., 2003).

Concerning the process of injury initiation, Cheung et al. discussed delayed-onset muscle soreness (DOMS) as a common consequence of unaccustomed eccentric exercise; however, DOMS should be interpreted as an acute symptom model and not as a clinical synonym for chronic skeletal muscle OUI (Cheung et al., 2003). Eccentric-load-induced microdamage can disrupt sarcolemmal integrity, leading to the release of intracellular substances like myoglobin and creatine kinase into the extracellular environment. Simultaneously, damage-associated molecular patterns (DAMPs, such as ATP and heat shock proteins) are produced, acting as “signal triggers” to initiate subsequent pathological responses.

An acute inflammatory response is quickly triggered within hours of the injury, serving both to clear damage and initiate repair. According to Tidball (2005), inflammation after an acute muscle injury is more than just a “destructive reaction.” Initially, neutrophils are drawn to the site of injury by chemokines like IL-8, where they remove necrotic muscle fiber debris through phagocytosis. Subsequently, macrophages shift from a pro-inflammatory M1 state to a pro-repair M2 state, producing cytokines like transforming growth factor-beta (TGF-β) and insulin-like growth factor-1 (IGF-1). This helps to prevent excessive inflammatory reactions, thereby avoiding further harm, and creates a microenvironment that supports subsequent healing (Tidball, 2005). Peake and colleagues (2017) discovered through cellular mechanism analysis that this acute inflammatory response is “short-term,” typically reaching its peak 48–72 h after injury and then gradually diminishing. The severity is closely related to the level of damage. A delayed or inadequate inflammatory response may lead to the incomplete removal of necrotic debris, thus hindering subsequent repair (Peake et al., 2017).

The repair of acute injury depends on the coordinated regulation of molecular signals and cellular responses. Warren et al. (2007), using gene expression evidence in mouse models, reported that within 12–24 h after acute muscle injury, the expression of inflammation-associated genes, such as NF-κB and IL-6, and repair-associated genes, such as MyoD and IGF-1 receptor, is substantially increased (Warren et al., 2007). This dynamic change in gene expression may promote satellite cell activity and support early repair response. Satellite cells are activated, proliferate, and differentiate from a quiescent state into myogenic precursor cells. These precursor cells gradually fuse with damaged myofibers to support structural repair (Warren et al., 2007). Summan et al. (2003) further reported the increased expression of inflammatory mediator genes, including MMP-9 and TNF-α receptor-related genes, after acute injury. These mediators may facilitate immune cell recruitment, extracellular matrix remodeling, satellite cell migration, and myogenic differentiation, thereby supporting an organized repair process (Summan et al., 2003).

In summary, acute eccentric-exercise-induced muscle damage provides a useful model for understanding the early sequence of myofiber disruption, inflammatory cell recruitment, satellite cell activation, and gene expression changes involved in repair. However, this acute model should not be interpreted as fully equivalent to chronic skeletal muscle OUI, which is shaped by repeated loading, insufficient recovery, and maladaptive remodeling.

##### 3.2.1.2 Chronic injury

Chronic skeletal muscle OUI is a load-related pathological state in which repeated microdamage and insufficient recovery gradually disrupt the balance between tissue damage, regeneration, and remodeling (Aicale et al., 2018). Unlike acute traumatic injury, chronic skeletal muscle OUI usually lacks a single initiating event and instead develops through repeated loading, insufficient recovery, and reduced tissue adaptability (Aicale et al., 2018).

A central feature of chronic skeletal muscle OUI is that the rate of accumulated microdamage exceeds the capacity for effective repair. Aicale et al. (2018) reported that excessive load, inadequate recovery, or abrupt fluctuations in load due to poorly planned training progression (e.g., a sudden spike in training volume) substantially increased the risk of chronic injury in different tissues, comprising bone, muscle, and tendon (Aicale et al., 2018). In adolescent athletes, Brenner et al. reported that unduly high training loads aligned with insufficient recovery not only increase the incidence of chronic injuries but also predispose individuals to overtraining syndrome and sports burnout, building a vicious cycle of “damage accumulation–decreasing repair capacity” (Brenner et al., 2024). Gabbett (2016), revealing the “training–injury prevention paradox,” further defined the load regulation threshold.

Rapid increases in training load have been associated with a higher risk of injury in some athletic populations, but the proposed thresholds vary across sports, monitoring methods, and individual tolerance. Therefore, training load indices should be interpreted as risk management tools rather than universal diagnostic cutoffs for skeletal muscle OUI (Soligard et al., 2016). This aligns with the findings of Kibler et al. (1992), who reported that long-term overtraining can cause abnormal adaptations in the muscle–tendon system, such as an increased proportion of fast-twitch fibers and altered tendon collagen structure, further decreasing the tissue’s damage tolerance threshold (Kibler et al., 1992).

San Emeterio et al. (2021), using animal models, reported that the core mechanism of chronic muscle and tendon injury involves persistent low-grade inflammation suppressing repair function. A chronic inflammatory environment substantially downregulates the expression of core differentiation factors (e.g., MyoD) in satellite cells, impairing muscle fiber regeneration. Simultaneously, it activates fibroblasts excessively, causing abnormal extracellular matrix remodeling (e.g., increased proportion of type III collagen, decreased proportion of type I collagen), thereby reducing tissue elasticity and increasing stiffness (San Emeterio et al., 2021). Clinically, as mentioned by Weerapong et al. (2005), such patients may present with activity-related pain and persistent dull pain at rest, with pain duration increasing as the condition progresses (Weerapong et al., 2005). Evidence from chronic tendinopathy is discussed only as adjacent musculoskeletal context and should not be interpreted as direct evidence for skeletal muscle OUI (Garrick, 2011). Chronic skeletal muscle OUI should instead be framed around repeated myofiber microdamage, insufficient recovery, impaired regeneration, persistent low-grade inflammation, and maladaptive ECM remodeling (Aicale et al., 2018; San Emeterio et al., 2021; Wohlgemuth et al., 2023).

Acute eccentric injury and chronic skeletal muscle OUI differ in temporal pattern, tissue adaptation, inflammatory dynamics, and repair outcome. Acute eccentric injury is generally associated with a discrete high-strain event and a time-limited inflammatory response, whereas chronic skeletal muscle OUI is associated with recurrent microdamage, insufficient recovery, persistent low-grade inflammation, and progressive impairment of repair capacity (Peake et al., 2017; Aicale et al., 2018; San Emeterio et al., 2021). A proposed pathological sequence for chronic skeletal muscle OUI is as follows: load–recovery imbalance → recurrent microdamage → persistent low-grade inflammation → impaired regeneration → maladaptive remodeling → functional decline (Aicale et al., 2018; San Emeterio et al., 2021; Wohlgemuth et al., 2023). This framework supports rehabilitation strategies that prioritize load control, modulation of excessive inflammation, and restoration of effective repair.

#### 3.2.2 Cellular responses and structural damage in skeletal muscle

**Figure 2:**
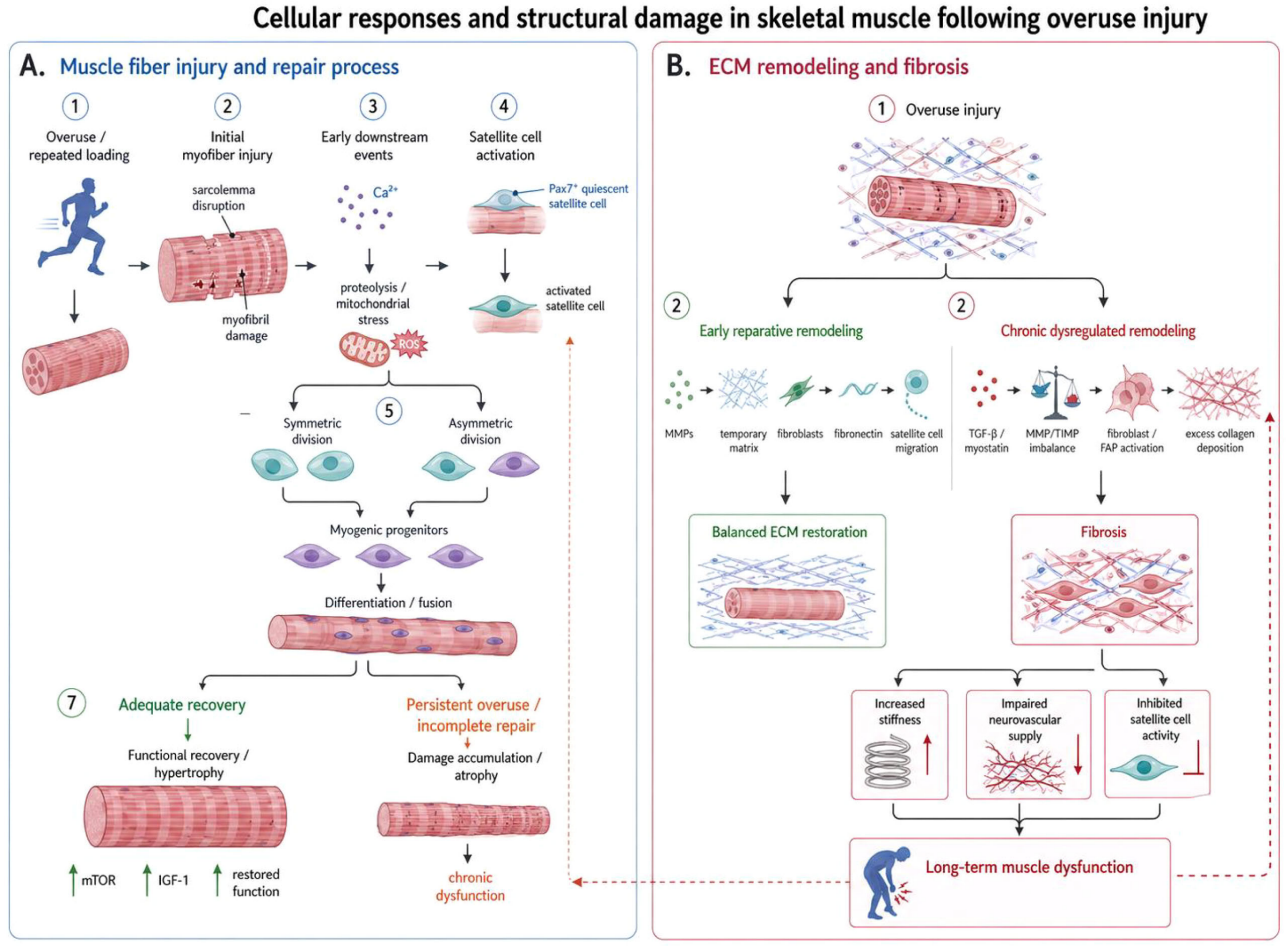
Muscle fiber injury, satellite cell repair, and ECM remodeling. **(A)** Muscle fiber injury and satellite-cell repair process after repeated loading, including myofiber disruption, calcium influx, metabolic stress, and satellite-cell activation. **(B)** ECM remodeling and fibrosis. Successful repair requires coordinated satellite-cell proliferation, differentiation, and fusion. Regulated ECM remodeling supports cell migration and force transmission, whereas dysregulated remodeling may promote fibrosis, stiffness, and impaired regeneration.

The cellular response of skeletal muscle to overuse load is a complex dynamic process involving the damage and repair of muscle fibers themselves as well as the remodeling of extracellular matrix. These two processes interact with each other and jointly determine the outcome of the injury. **Figure 2** summarizes the cellular responses and structural changes associated with skeletal muscle OUI.

##### 3.2.2.1 Muscle fiber injury and repair process

As the basic contractile units of skeletal muscle, myofibers are central to force generation, movement efficiency, and functional recovery after injury. Repeated overuse may disturb myofiber homeostasis and initiate structural, metabolic, and inflammatory responses. The efficiency and coordination of repair processes are important determinants of recovery (Schiaffino, 2010). This mechanism can be divided into three stages—”damage initiation,” “repair activation,” and “functional recovery/damage accumulation”—each stage is controlled by different molecular and cellular regulatory processes.

Muscle fiber injury caused by overuse is a cascade pathological reaction when the biomechanical tension exceeds the physiological tolerance threshold of the fiber. Tidball and Villalta (2010) reported that when the muscle is subjected to repeated or high-intensity biomechanical loads, the initial event is the focal disruption of the sarcolemma. This is followed by destruction of intracellular homeostasis, defined by abnormal activation of protein degradation pathways (e.g., the ubiquitin–proteasome system) and hindered mitochondrial function (e.g., excessive reactive oxygen species generation, energy supply failure). These changes jointly cause the structural disintegration and functional decline of the muscle fiber (Tidball and Villalta, 2010). Tidball (2011) further distinguished pathological differences between injury types: damage from eccentric contractions mainly involves direct destruction of myofibrils (e.g., Z-line disorganization, actin–myosin cross-bridge rupture), whereas other acute injuries, such as heat stress or contusion, are defined by extensive sarcolemmal damage (Tidball, 2011). Regardless of the injury type, calcium influx coming from membrane rupture is a critical downstream event—excessive calcium activates hydrolytic enzymes, such as:

phospholipases and proteases, promoting fiber degradation, while also functioning as a signaling molecule to start subsequent repair programs (Tidball, 2011).

Muscle fiber repair depends on the precise regulation of muscle satellite cells (skeletal muscle stem cells), which is the core mechanism of regulating skeletal muscle tissue homeostasis (Kong et al., 2024). As early as 1971, Moss and Leblond first reported in rats that satellite cells are the main source of myoblast nuclei during muscle fiber repair—following injury, satellite cells, generally quiescent between the basal lamina and sarcolemma, are swiftly activated (Moss and Leblond, 1971). Dumont et al. (2015) further clarified the repair mechanisms of satellite cells: activated satellite cells are heterogeneous; a small subset (satellite stem cells) sustains the stem cell pool via symmetric division, while the majority face asymmetric division to produce myogenic progenitor cells. These progenitors then proliferate and migrate to the injury site, finally engaging in repair through two processes—fusing with one another to form new myotubes or fusing with damaged myofibers to replenish myonuclei and synthesize new contractile proteins (e.g., actin, myosin), thereby supporting the structural reconstruction of the myofiber (Dumont et al., 2015). Pallafacchina et al. (2013) added that the increase in myonuclei induced by satellite cells is not only vital for acute injury repair but also fundamental to muscle fiber hypertrophy during long-term exercise adaptation—an enhanced myonuclear number increases the fiber’s protein synthesis capacity, offering material support for structural remodeling (PallafacChina et al., 2013). Striedinger et al. (2021) established a method for the isolation and purification of human satellite cells through technological progress, providing an experimental basis for further analysis of its repair function (Striedinger et al., 2021).

In the adaptive stage after repair, long-term repeated training can promote muscle fiber hypertrophy, thereby contributing to functional adaptation. Early research by Tesch (1988) found that long-term high-load endurance training can significantly improve the cross-sectional area of muscle fibers, and this hypertrophy is more obvious in fast twitch fibers (Tesch, 1988). Schoenfeld (2010) further proposed that biomechanical tension, muscle damage, and metabolic stress are the three main factors driving muscle hypertrophy—mechanical tension activates force-sensitive signaling pathways within the muscle (e.g., mTOR), muscle damage induces the generation of inflammatory factors (e.g., IGF-1), and metabolic stress enhances anabolic mechanisms via metabolites, such as lactate. These factors synergistically improve muscle protein synthesis (Schoenfeld, 2010). Subsequent evidence further refined this mechanism: Schoenfeld et al. (2015) reported that both high-load and low-load resistance training can drive hypertrophy, but high-load training is more positive for enhancing strength (Schoenfeld et al., 2015); Haun et al. (2019) and Andersen and Aagaard (2010), in that order, reported that hypertrophy driven by high-volume resistance training is mainly attributable to sarcoplasmic hypertrophy and that muscle fiber adaptation in high-level athletes involves both fiber type transition and size increase (Andersen and Aagaard, 2010; Haun et al., 2019).

The repair ability of muscle fibers is not unlimited. If the repair mechanism is incomplete (e.g., because of insufficient satellite cell activity, imbalances in protein synthesis) or if the muscle remains in a state of persistent overuse (lacking adequate recovery time), damage increasingly accumulates, eventually causing chronic injury. Evidence from spinal-cord-injury-related compensatory overuse suggests that incomplete repair and chronic abnormal loading may contribute to persistent muscle atrophy and degeneration, although this model should be interpreted as indirect rather than specific evidence for skeletal muscle OUI (Yilmaz et al., 2005). This result also emphasizes that adequate recovery time and a stable repair environment are the key factors to avoid the chronicity of muscle fiber injury.

##### 3.2.2.2 Extracellular matrix remodeling and fibrosis

Evidence on extracellular matrix (ECM) remodeling in chronic skeletal muscle OUI remains limited, and several studies discussed in this section come from related but indirect models of muscle injury, fibrosis, volumetric muscle loss, burn injury, rotator cuff-associated muscle degeneration, or osteoarthritis-associated muscle changes (Borisov et al., 2000; Gumucio et al., 2013; Gigliotti et al., 2017; Brightwell et al., 2020; Corona et al., 2020; Wohlgemuth et al., 2023; Wei et al., 2024; Yu et al., 2025). These models are useful for understanding fibrosis, matrix stiffness, impaired vascularization, and regenerative failure, but they should not be considered equivalent to skeletal muscle OUI. ECM is an important structural and physiological component of skeletal muscle. It not only offers biomechanical support for muscle fibers but also participates in tissue regeneration post-injury by modulating cell signaling and constructing a reparative microenvironment. The balance of ECM remodeling can influence repair outcomes: regulated remodeling may support regeneration, whereas persistent dysregulation may contribute to fibrosis, increased stiffness, and impaired muscle function (Wohlgemuth et al., 2023).

The skeletal muscle ECM is organized into the epimysium, perimysium, and endomysium. Its key components are collagen (mainly types I and III), proteoglycan, and glycoprotein. Its organization directly influences the biomechanical properties and regenerative capacity of skeletal muscle (Wohlgemuth et al., 2023). Under normal physiological conditions, the ECM promotes lateral transmission of contractile force via the orderly arrangement of collagen fibers while protecting the sarcolemma from contraction-induced biomechanical damage. Following injury, the ECM initially undergoes a reparative remodeling program to support regeneration through several mechanisms: in the early stage, the activity of matrix metalloproteinases (MMPs; e.g., MMP-1, MMP-9) increases, degrading damaged collagen fibers and matrix proteins to clear a path for the migration of inflammatory cells (e.g., macrophages) and satellite cells. Simultaneously, fibroblasts are activated to synthesize new collagen (primarily type III collagen, which is easily degradable and conducive to tissue shaping) and growth factors (e.g., FGF, IGF-1), constructing a temporary reparative matrix (Brightwell et al., 2020). ECM components (e.g., fibronectin), by binding to cell surface integrins, activate proliferation and differentiation signals (e.g., the PI3K–AKT pathway) in satellite cells and guide the recruitment of inflammatory cells to the injury site, coordinating the timing of “damage clearance and regeneration initiation” (Wohlgemuth et al., 2023). Wohlgemuth et al. (2023) stressed that the key to early reparative remodeling lies in the “dynamic balance between collagen synthesis and degradation”—if this balance is maintained, the repaired ECM progressively restores its original structural orderliness, ensuring muscle elasticity and contractile function. However, continuous overuse disturbs this balance and leads to pathological remodeling (Wohlgemuth et al., 2023).

Long-term repeated overuse injury can lead to uncontrolled ECM remodeling process, which changes from “repair-oriented” state to “fibrosis-oriented” state. Its core mechanisms include the continuous activation of fibrogenic signals, imbalance of collagen metabolism, and abnormal proliferation of fibroblasts. Persistent injury leads to chronic high expression of fibrogenic factors such as transforming growth factor-β (TGF-β) and myostatin (Gumucio et al., 2013). In an indirect burn-related muscle injury model, Brightwell et al. (2020) observed increased myostatin signaling and expansion of fibrogenic/adipogenic progenitors during post-injury remodeling (Brightwell et al., 2020). These progenitor cells are widely differentiated into fibroblasts, providing a cellular basis for excessive collagen deposition (Brightwell et al., 2020). In a rat myofascial trigger point model, Fang et al. (2023) suggested that TGF-β1–Smad2/3 signaling may contribute to fibrosis-related remodeling and that tissue stiffness may be associated with collagen deposition (Fang et al., 2023). In normal repair, the ratio of MMPs to tissue inhibitors of metalloproteinases (TIMPs) remains stable, ensuring synchronized collagen degradation and synthesis. During overuse, however, TIMP expression (especially TIMP-1) is substantially elevated, suppressing the collagen-degrading activity of MMPs. Brightwell et al. (2020) observed increased TIMP-1 expression in mouse muscle after burn injury, causing a collagen degradation rate lower than the synthesis rate, thus causing net collagen accumulation (peaking at day 14 and remaining high at day 21) (Brightwell et al., 2020). In addition, excessive remodeling is consistent with the change of collagen type. The proportion of type III collagen (repair related, flexible) decreased, while the proportion of type I collagen (tough, but less elastic) increased, resulting in ECM hardening (Wohlgemuth et al., 2023). Long-term overuse deprives the muscle of sufficient recovery time, so ECM repair is incomplete after each injury attack. Damaged and disorganized collagen may accumulate together with newly synthesized matrix components. In muscle tissue from patients with rotator cuff injuries, Gigliotti et al. (2017) observed fibrosis, reduced vascular density, and altered fiber characteristics, providing indirect evidence that chronic musculoskeletal injury can be associated with adverse muscle remodeling (Gigliotti et al., 2017).

ECM fibrosis may contribute to persistent muscle dysfunction by increasing tissue stiffness and altering the regenerative microenvironment. In fibrotic tissues, collagen fibers are disordered and over-crosslinked, resulting in significantly reduced muscle elasticity and increased stiffness. Evidence from indirect models, including volumetric muscle loss, burn-related muscle injury, rotator-cuff-associated muscle degeneration, and osteoarthritis-associated muscle changes, supports the broader principle that excessive fibrosis can increase tissue stiffness, impair vascular and neural support, and restrict regenerative cell migration. However, these models differ substantially from chronic skeletal muscle OUI in etiology, injury magnitude, and repair environment. Therefore, they should be used to generate mechanistic hypotheses rather than to establish causal claims specific to skeletal muscle OUI (Borisov et al., 2000; Gumucio et al., 2013; Gigliotti et al., 2017; Brightwell et al., 2020; Corona et al., 2020; Wohlgemuth et al., 2023; Wei et al., 2024; Yu et al., 2025).

Overall, skeletal muscle ECM remodeling has context-dependent effects: early and well-regulated remodeling may support regeneration, whereas persistent or dysregulated remodeling may contribute to fibrosis and impaired repair. Fibrosis may reduce muscle elasticity, disturb force transmission, impair neurovascular support, and limit regenerative efficiency. Therefore, maintaining balanced ECM remodeling and limiting excessive fibrosis may be important goals in the prevention and rehabilitation of chronic skeletal muscle OUI.

#### 3.2.3 Inflammatory responses and oxidative stress in skeletal muscle OUI

**Figure 3:**
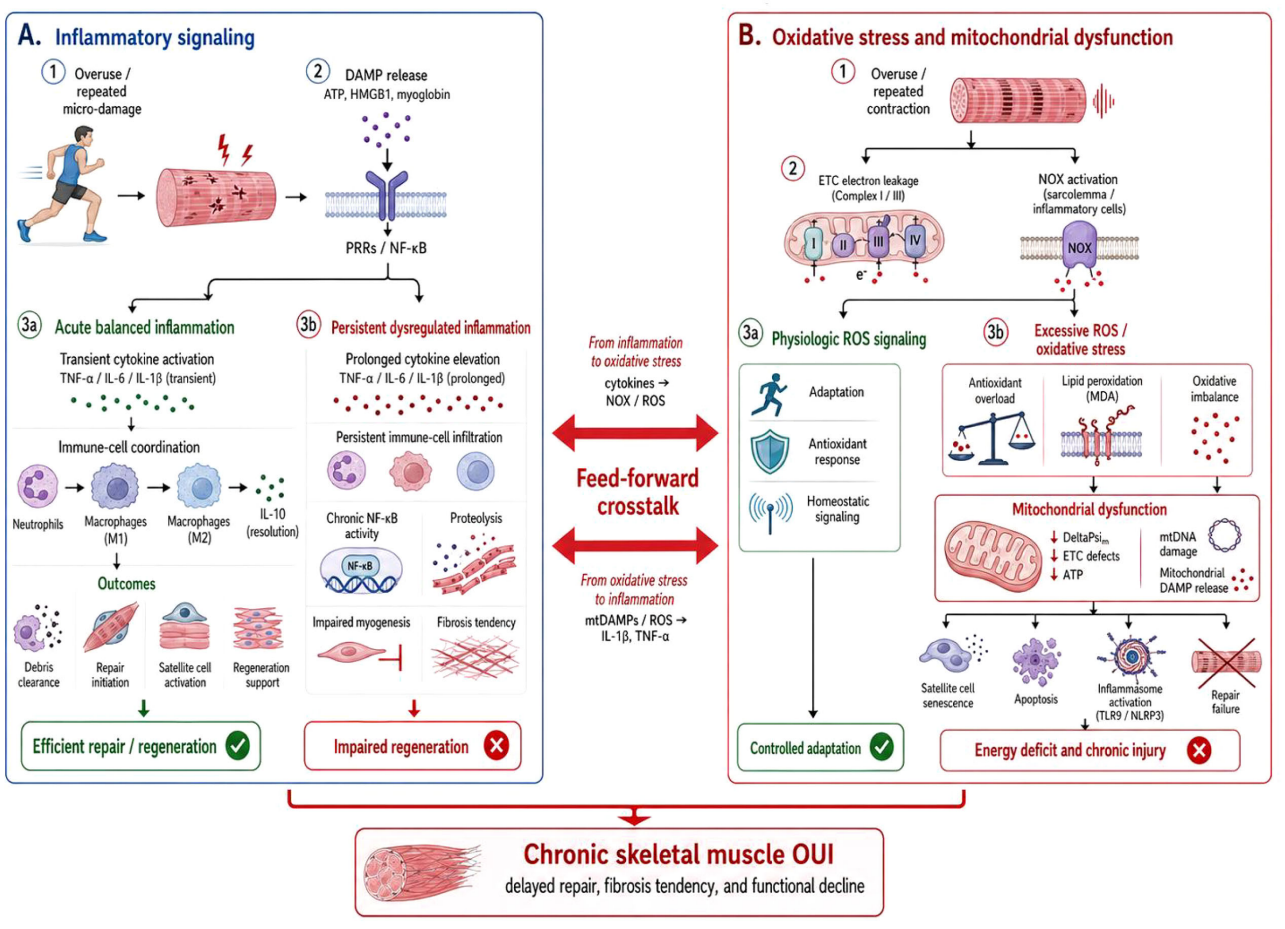
Inflammation, oxidative stress, and mitochondrial dysfunction in skeletal muscle OUI. **(A)** Inflammatory signaling, in which acute cytokine activation supports debris clearance and repair, whereas persistent signaling may impair regeneration. **(B)** Oxidative stress and mitochondrial dysfunction, including excessive ROS generation, impaired ATP production, and mitochondrial DAMP release. The red bidirectional arrows indicate feed-forward crosstalk between chronic inflammation and oxidative stress.

Inflammation and oxidative stress should not be presented as uniformly harmful because transient inflammatory signaling and low-to-moderate reactive oxygen species can support debris clearance, adaptation, and repair (Tidball and Villalta, 2010; Peake et al., 2017; Tu and Li, 2023). Pathology is more likely to emerge when these responses become excessive, prolonged, spatially dysregulated, or uncoupled from effective regeneration (Tidball and Villalta, 2010; Peake et al., 2017; Tu and Li, 2023). The pathological progression of skeletal muscle injury caused by overuse is not only controlled by structural damage to muscle fibers and extracellular matrix but also by the dynamic interaction between inflammatory response and oxidative stress. These two mechanisms are closely related and form a regulatory network that determines repair outcomes: timely acute inflammatory response is crucial for clearing damaged tissue and activating regeneration programs, while unresolved chronic inflammation shifts the microenvironment towards fibrosis and functional decline. When excessive or unresolved, oxidative stress may damage cellular structures, impair mitochondrial function, disrupt energy homeostasis, and reinforce inflammatory signaling through feed-forward loops. **Figure 3** summarizes inflammation, oxidative stress, and mitochondrial dysfunction in skeletal muscle OUI.

##### 3.2.3.1 Activation and function of inflammatory cytokines

Following skeletal muscle injury, inflammatory cytokines participate in damage recognition, debris clearance, repair initiation, and tissue remodeling. Their effects are time-dependent and context-dependent: moderate activation during the acute phase can support repair, whereas persistent activation during chronic maladaptation may impair regeneration. This mechanism depends on the precise signal dialogue between muscle cells and immune cells (such as macrophages and neutrophils). Core inflammatory cytokines (such as TNF-α, IL-6, and IL-1β) regulate the repair process through different processes (Tidball and Villalta, 2010).

Following overuse-driven muscle fiber micro-damage, the activation of inflammatory cytokines starts with the generation of damage-associated molecular patterns (DAMPs) and the recruitment of immune cells. After sarcolemmal disruption, intracellular DAMPs (e.g., ATP, heat shock proteins, myoglobin) are released into the extracellular space, where they bind to pattern recognition receptors (PRRs) on immune cells (e.g., macrophages), triggering downstream signaling pathways (e.g., NF-κB) and inducing the transcription of inflammatory cytokines (Tu and Li, 2023). At the same time, the complement system is activated, further amplifying the immune response and promoting the migration of neutrophils and macrophages to the injury site. Inflammatory cytokines are mainly secreted by two cell types: first, the damaged myofibers themselves, which synthesize TNF-α and IL-6 directly in response to biomechanical stress and DAMP stimulation; second, infiltrating immune cells, notably macrophages. In the early phase of injury (12–24 h), M1 macrophages predominate, generating large amounts of pro-inflammatory factors. At the later stage (48–72 h), they change to M2 phenotype and produce anti-inflammatory factors (such as IL-10), thereby contributing to the temporal regulation of inflammation (Tidball and Villalta, 2010).

During the acute inflammatory phase (24–72 h post-injury), pro-inflammatory cytokines, such as TNF-α and IL-6, exert protective roles in clearing damaged cellular debris and starting muscle regeneration, constituting an indispensable part of the repair program. TNF-α accelerates the clearance of necrotic muscle fiber fragments and denatured proteins by enhancing the phagocytic activity of neutrophils and M1 macrophages, thus building space for subsequent repair. Simultaneously, TNF-α can drive macrophages to generate nitric oxide (NO), which aids in breaking down stubborn injured tissue; nevertheless, excessive NO can cause secondary damage, necessitating tight modulation of its expression (Tidball and Villalta, 2010). IL-6 is a core factor linking inflammation and muscle repair. On the one hand, it promotes the proliferation of satellite cells (muscle stem cells) by activating the STAT3 signaling pathway, providing sufficient muscle-derived progenitor cells for muscle fiber repair. On the other hand, it can upregulate the expression of hepatocyte growth factor (HGF) and insulin-like growth factor-1 (IGF-1) and promote the differentiation of muscle-derived progenitor cells (Liu et al., 2017). Liu et al. (2017), using macrophage depletion experiments in mice, reported that the absence of macrophages (and thus their secreted TNF-α and IL-6) led to the downregulation of myogenic regulatory factors (MyoD, myogenin) and decreased levels of repair factors, such as HGF and IGF-1, directly delaying muscle fiber regeneration. This suggests that appropriately timed inflammatory signaling is required for efficient muscle repair (Liu et al., 2017). Inflammatory cytokines also coordinate the repair phase by regulating the phenotypic transition of immune cells. TNF-α and IL-6 maintain the pro-inflammatory phenotype of M1 macrophages in the early stage of injury and ensure positive damage clearance. Subsequently, by activating anti-inflammatory factors such as IL-10, M1 macrophages were induced to transform into M2 macrophages, and an anti-inflammatory microenvironment conducive to muscle fiber differentiation was established (Peake et al., 2017). Peak et al. (2017) reported that the destruction of this time switch (for example, the persistence of M1 macrophages) would delay the transition of myogenesis from “proliferation phase” to “differentiation phase,” thus leading to repair stagnation (Peake et al., 2017).

When OUI occurs repeatedly and the recovery time is insufficient, inflammatory cytokine signaling may lose its temporal resolution and enter a state of continuous high expression. Their core mechanism is that chronic low-grade inflammation inhibits the repair pathway and induces tissue degeneration. Long-term high levels of TNF-α and IL-1β upregulate the expression of p21 and other cell cycle inhibitors in satellite cells by activating the p38 MAPK pathway and inhibit their proliferation and differentiation. At the same time, chronic inflammation reduces the expression of IGF-1 receptor in satellite cells, making them insensitive to repair signals (such as IGF-1) and ultimately reducing the ability of muscle fiber regeneration (Tu and Li, 2023). Persistent inflammation may sustain elevated cytokine signaling, neutrophil activation, ROS production, and protease release. This not only damages the remaining healthy muscle fibers but also disturbs vascular endothelial function, decreasing the oxygen and nutrient supply to the injured area, thus building a vicious cycle of “inflammation–ischemia–re-injury” (Tu and Li, 2023). Evidence from chronic inflammatory myopathies suggests that neutrophil activation and NET formation can contribute to myofiber degeneration and fibrosis; however, this evidence is indirect for skeletal muscle OUI (Torres-Ruiz et al., 2023). Chronic inflammatory factors (especially TGF-β1 and IL-6) activate the fibroblasts’ excessive proliferation and induce them to synthesize large amounts of type III collagen. This can lead to excessive deposition and fibrosis of extracellular matrix (ECM)—a process that further hinders muscle fiber regeneration, reduces muscle elasticity, and exacerbates chronic pain (Liu et al., 2017).

The effects of inflammatory cytokines in skeletal muscle OUI are context-dependent and vary according to timing, concentration, cellular source, and tissue microenvironment. In the acute phase (24–72 h after injury), pro-inflammatory factors such as TNF-α and IL-6 promote repair by clearing damaged tissue and activating satellite cells. However, if overload and inadequate recovery persist, resulting in the sustained high expression of these factors, they will transform into chronic inflammation, inhibit regeneration, and induce fibrosis. These findings support the concept that inflammation should be interpreted in a timing-sensitive manner: early inflammatory signaling may support repair, whereas persistent inflammatory activation may impair regeneration and promote maladaptive remodeling.

##### 3.2.3.2 Oxidative stress and mitochondrial dysfunction

Oxidative stress is a biologically plausible contributor linking repeated mechanical loading to cellular dysfunction in skeletal muscle OUI. Excessive reactive oxygen species (ROS) produced by repeated contraction not only directly damages the cell structure but also targets mitochondria, the main organelles responsible for oxidative ATP production, thus contributing to progressive functional impairment. This may create a feed-forward cycle of oxidative stress, mitochondrial impairment, energy deficit, and delayed repair. This mechanism is supported by evidence from acute exercise, metabolic stress, cell culture, and hereditary myopathy models, but its causal contribution to chronic skeletal muscle OUI requires further direct investigation (Powers and Jackson, 2008).

Muscle overuse leads to oxidative stress, with reactive oxygen species mainly originating from two principal pathways that synergistically enhance the injury process: (1) Leakage from the mitochondrial respiratory chain occurs as mitochondria boost aerobic respiration to satisfy energy needs during extended muscle contraction. However, imbalance in the electron transport chain (ETC), for instance, electron leakage from complex I or III, results in the generation of large quantities of reactive oxygen species (ROS) such as O_2_^-^· and H_2_O_2_. Powers and Jackson (2008) pointed out in their review that mitochondrial ROS generation rates during strenuous exercise can be three to five times higher than at rest. ROS preferentially attack mitochondria themselves, as their membranes are rich in polyunsaturated fatty acids, making them susceptible to lipid peroxidation. This forms toxic products, such as malondialdehyde (MDA), which further destroys the structure of the respiratory chain (Powers and Jackson, 2008). (2) Activation of NADPH oxidase: NADPH oxidase (NOX) in the sarcolemma and inflammatory cells (e.g., neutrophils, macrophages) is another significant ROS source. Following overuse-driven sarcolemmal micro-damage, NOX is activated by DAMPs (e.g., ATP, HMGB1), continuously generating ROS to engage in damage clearance. Nevertheless, excessive ROS overwhelm the compensatory capacity of the antioxidant system (e.g., SOD, GSH-Px), causing “oxidative imbalance” (Tu and Li, 2023). It is worth noting that ROS are not simply “toxic molecules.” Low concentrations of ROS can act as signaling molecules modulating cell proliferation (e.g., activating MAPK pathways) and gene expression (e.g., upregulating antioxidant enzyme genes). Nevertheless, the ROS “burst” triggered by overuse disturbs this balance, shifting towards pathological damage (Powers and Jackson, 2008).

Mitochondria are particularly vulnerable to excessive oxidative stress, which may impair membrane integrity, respiratory enzyme activity, ATP production, and mitochondrial DNA stability. Evidence from exercise, cell, metabolic stress, toxic exposure, and hereditary myopathy models suggests that excessive oxidative stress can impair mitochondrial membrane potential, respiratory chain activity, ATP production, and mitochondrial DNA stability (Powers and Jackson, 2008; Ambrosio et al., 2014; Jaiswal et al., 2015; Bonato et al., 2024). These changes may compromise the energetic support required for satellite cell activation, myofiber repair, and restoration of contractile function (Powers and Jackson, 2008; Ambrosio et al., 2014; Dumont et al., 2015). However, most of this evidence derives from indirect models rather than from chronic skeletal muscle OUI—for example, high-fructose-treated myotubes, arsenic exposure models, and Duchenne muscular dystrophy models provide mechanistic insight into mitochondrial vulnerability, but they do not directly establish the magnitude or causal role of mitochondrial dysfunction in chronic skeletal muscle OUI (Ambrosio et al., 2014; Jaiswal et al., 2015; Bonato et al., 2024). Therefore, these findings should be interpreted as mechanistic support for a potential oxidative stress–mitochondrial dysfunction axis rather than as direct clinical evidence for skeletal muscle OUI.

Mitochondrial dysfunction may influence skeletal muscle repair not only by limiting energy supply but also by affecting inflammatory signaling, apoptosis, and satellite cell function. Impaired mitochondria may release mitochondrial damage-associated molecular patterns, including mitochondrial DNA and N-formyl peptides, which can activate inflammatory pathways and reinforce cytokine production. These mechanisms are biologically plausible, but current evidence remains largely indirect for chronic skeletal muscle OUI (Powers and Jackson, 2008; Ambrosio et al., 2014; Jaiswal et al., 2015; Choi, 2018; Bonato et al., 2024).

Antioxidant or mitochondria-targeted strategies may have future relevance as adjunctive approaches, but their clinical efficacy in chronic skeletal muscle OUI remains unproven. Existing evidence from cell, oxidative stress, metabolic disease, cachexia, or pharmacological models is useful for hypothesis generation, but it should not be interpreted as direct therapeutic evidence for skeletal muscle OUI (Molinari et al., 2017; Choi, 2018). At present, such strategies should be discussed as experimental or exploratory rather than established rehabilitation interventions.

Overall, excessive oxidative stress may act as a pathological amplifier in skeletal muscle OUI by impairing mitochondrial function, energy homeostasis, and regenerative signaling. However, because much of the current evidence derives from acute exercise, metabolic stress, toxic exposure, hereditary myopathy, cachexia, or cell culture models, its relevance to chronic skeletal muscle OUI should be interpreted cautiously.

#### 3.2.4 Biomechanical factors: the influence of biomechanical stress on muscle

Repeated mechanical stress is a major external contributor to skeletal muscle OUI. Mechanical loading may contribute to skeletal muscle OUI through excessive strain, uneven stress distribution, fatigue-related loss of force control, and impaired coordination across the muscle–tendon–joint system (Taimela et al., 1990).

Excessive or poorly distributed mechanical loading can increase local strain within skeletal muscle, particularly when high training volume, insufficient recovery, fatigue-related loss of force control, or altered movement patterns are present. Evidence from broader musculoskeletal research indicates that external load carriage, abnormal joint mechanics, anatomical variation, and strength imbalance can redistribute stress across the lower limb. However, many of these data derive from tendon-, bone-, or joint-related outcomes rather than direct skeletal muscle OUI models. Therefore, biomechanical evidence should be interpreted as supporting a general load–tolerance framework rather than as definitive tissue-specific proof for skeletal muscle OUI (Taimela et al., 1990; Haggerty et al., 2014; Earl-Boehm et al., 2020).

**Figure 4:**
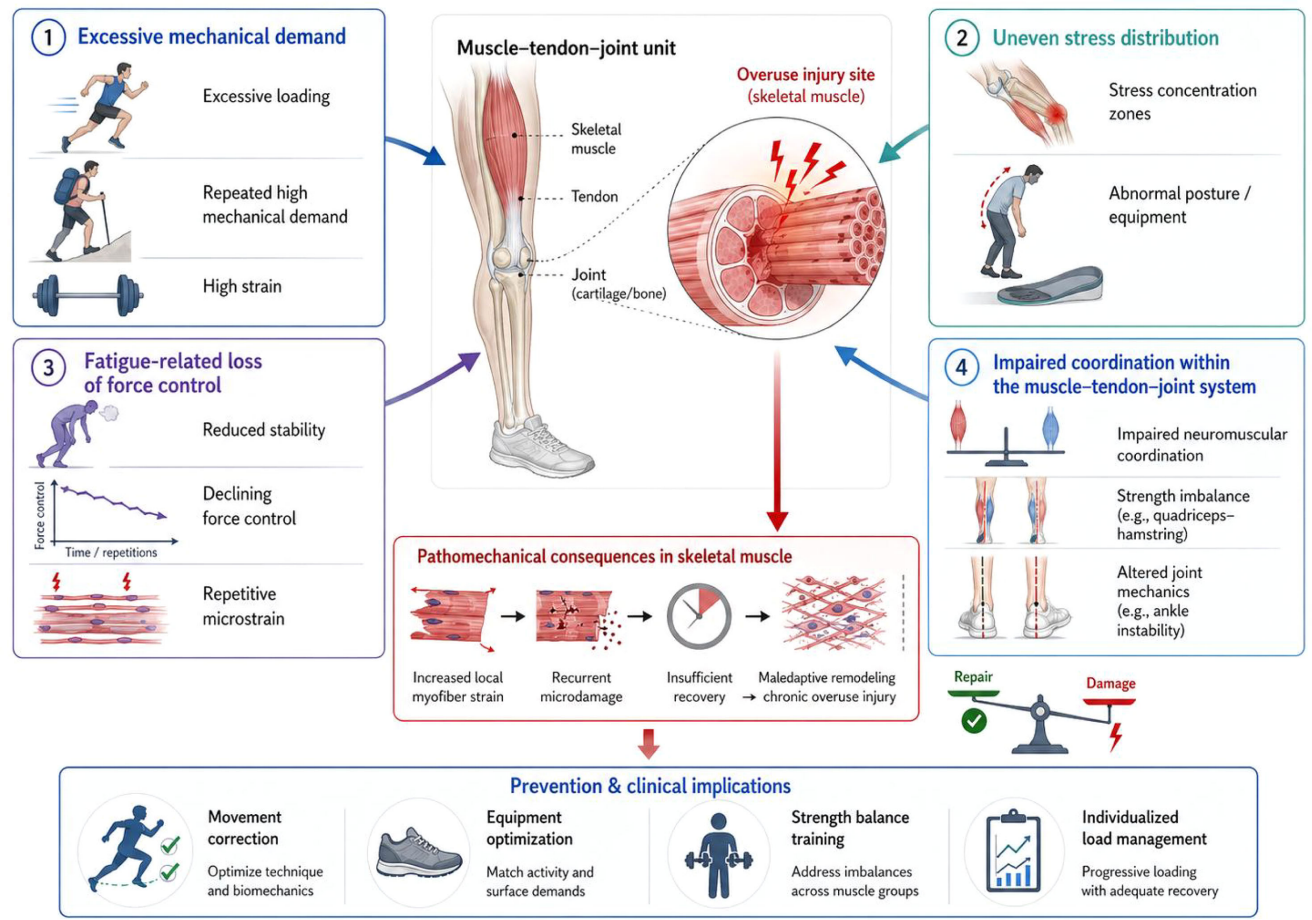
Biomechanical contributors to skeletal muscle OUI. Excessive mechanical demand, uneven stress distribution, fatigue-related loss of force control, and impaired neuromuscular coordination can increase local myofiber strain. These factors may interact with insufficient recovery to promote recurrent microdamage and maladaptive remodeling. Adjacent musculoskeletal tissues are shown only as secondary loading contexts.

In general, biomechanical contributors to skeletal muscle OUI include excessive loading, uneven stress distribution, and impaired coordination within the muscle–tendon–joint system. Prevention strategies should aim to restore biomechanical balance through movement correction, equipment optimization, strength balance training, and individualized load management. **Figure 4** summarizes the biomechanical contributors to skeletal muscle OUI.

### 3.3 Molecular pathways involved in skeletal muscle OUI

**Figure 5:**
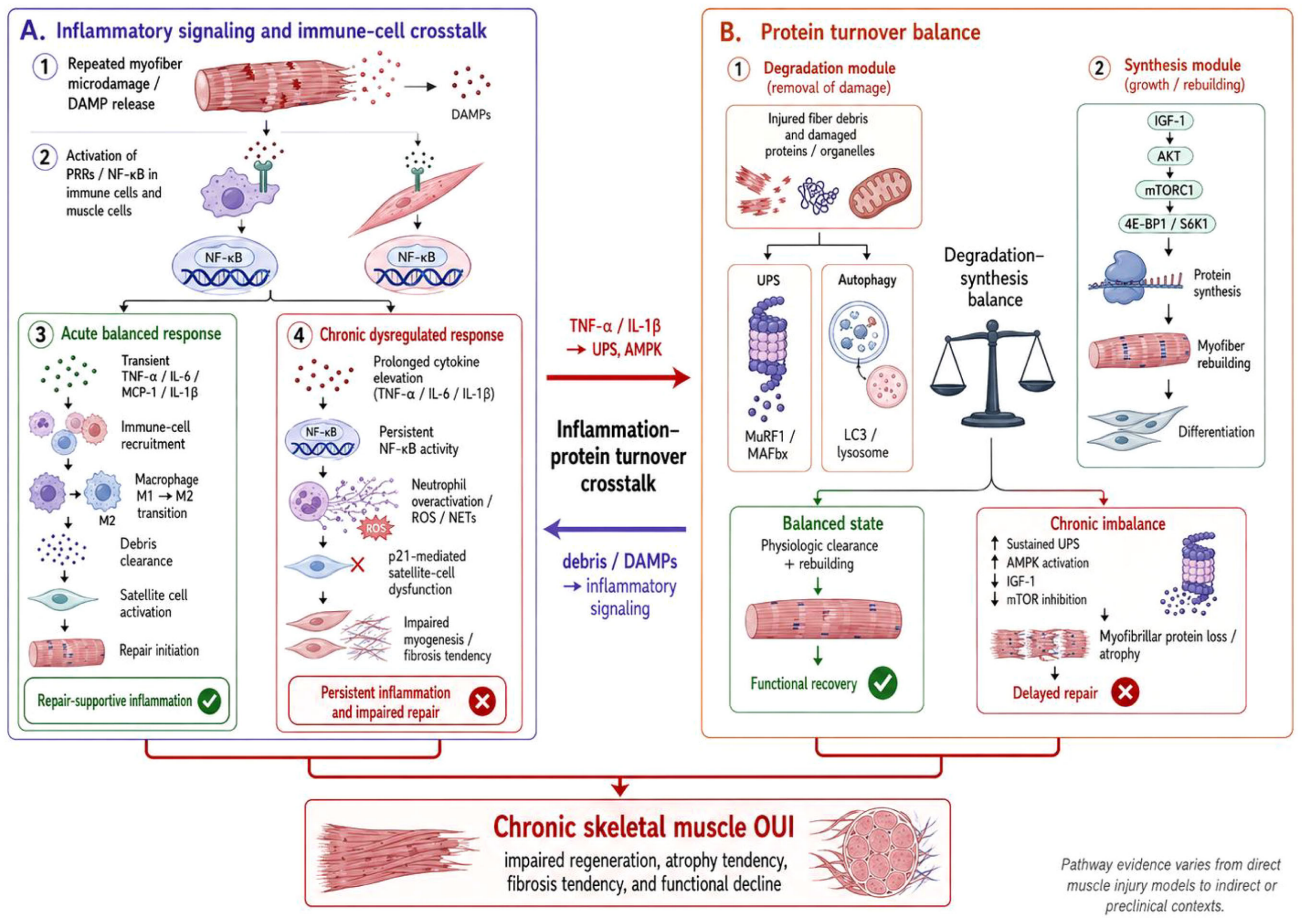
Inflammatory signaling and protein turnover in skeletal muscle OUI. Inflammatory cytokines participate in damage recognition, immune cell recruitment, and repair initiation, but persistent signaling may impair regeneration. Protein turnover reflects the balance between degradation pathways, including the ubiquitin–proteasome system and autophagy, and synthesis pathways, including mTOR signaling. Chronic imbalance may favor atrophy and delayed repair.

The development and repair of skeletal muscle OUI involve coordinated regulation of inflammatory, metabolic, myogenic, and remodeling pathways. The supporting evidence varies in directness, ranging from skeletal muscle injury and regeneration studies to indirect models of chronic tissue remodeling or experimental molecular intervention. Therefore, the following sections discuss each pathway with attention to whether the evidence is direct, indirect, preclinical, or extrapolative. **Figure 5** summarizes the core molecular signaling pathways involved in skeletal muscle OUI.

#### 3.3.1 Inflammatory signaling and immune-cell crosstalk

Pro-inflammatory cytokines are key signaling molecules linking damage recognition, immune cell recruitment, repair initiation, and tissue remodeling after skeletal muscle injury. Their role displays significant stage-dependent characteristics: in the acute injury phase, they promote repair by recruiting immune cells to clear necrotic tissue and activating muscle satellite cells (stem cells); nevertheless, if their expression remains persistently high, they transition to promoting chronic inflammation, which conversely exacerbates tissue damage. The effectiveness and outcome of muscle repair may depend partly on the timing and magnitude of this dual response (Tu and Li, 2023).

Pro-inflammatory cytokines, including TNF-α, IL-6, and MCP-1, are quickly activated within hours after an injury. Damage-associated molecular patterns (DAMPs; e.g., ATP, heat shock proteins) produced by injured muscle fibers bind to receptors on macrophages and mast cells, inducing the secretion of pro-inflammatory factors, such as TNF-α and IL-6. These factors recruit neutrophils and monocytes to the injury site through the bloodstream, starting a cascade of “immune cell infiltration and debris clearance.” Tu et al. (2023) reported that macrophages and neutrophils clear necrotic myofiber debris via phagocytosis while simultaneously generating secondary inflammatory factors (e.g., IL-8) to further amplify the immune response, thereby building “space” for subsequent regeneration. In addition, this mechanism stimulates myoblast fusion and angiogenesis, providing nutritional support for repair (Tu and Li, 2023). Although Perry et al. (1993) focused on central nervous system inflammation, their work determined macrophages as key cells in “debris clearance and inflammation regulation” post-injury—a mechanism equally applicable to skeletal muscle injury. In the acute phase, macrophages predominantly display the M1 (pro-inflammatory) phenotype, and their function depends on the maintained activation of pro-inflammatory factors (Perry et al., 1993). Chow et al. (2022) further found that proinflammatory cytokines can regulate the conversion of macrophage phenotype from M1 to M2 (repair phenotype), and this timely polarization is crucial for bridging the “clearance repair” stage (Chow et al., 2022).

Pro-inflammatory cytokines can contribute to the transition of satellite cells from quiescence to activation. Fu et al. (2015) showed that the integration of pro-inflammatory factors (such as IL-1α and TNF-α) can significantly improve the proliferation of satellite cells *in vitro*. Adding these factors to the culture system can make the satellite cells continuously expand for more than 20 generations and still repair multiple rounds of muscle injury after transplantation. This suggests that proinflammatory factors can maintain not only the activity of satellite cells but also the characteristics of stem cells (Fu et al., 2015). Joanisse et al. (2016) focused on the function of IL-6. The results showed that IL-6-deficient mice showed significantly reduced satellite cell proliferation and regeneration efficiency by 40% after muscle injury. IL-6 activates the STAT3 signaling pathway, upregulates the expression of MyoD (a core myogenic differentiation factor) in satellite cells, and promotes the transformation of satellite cells from proliferation to differentiation (Joanisse and Parise, 2016). It is suggested that proinflammatory cytokines are not only “inflammatory signals” but also direct “molecular switches” regulating myogenesis.

If excessive use leads to repeated injury, pro-inflammatory cytokine signaling may lose temporal resolution and enter a state of continuous high expression, shifting from a repair-supportive signal to a maladaptive inflammatory stimulus. Wang et al. (2021) reported that persistently high levels of TNF-α and IL-6 in chronic inflammation induce the excessive activation of immune cells, generating large amounts of reactive oxygen species (ROS) and lysosomal enzymes. This not only damages the remaining healthy muscle fibers but also weakens the function of vascular endothelium, resulting in ischemia and hypoxia at the injured site, thus forming a vicious cycle of “inflammation ischemia re-injury” (Wang et al., 2021). Castanheira et al. (2019) reported that in chronic inflammation, the maintained generation of neutrophil extracellular traps (NETs) by neutrophils suppresses satellite cell proliferation and differentiation. At the same time, chronically elevated TNF-α upregulated the expression of p21 (cell cycle inhibitor) in satellite cells and induced cell senescence and decreased regeneration ability (Castanheira and Kubes, 2019). Khansari et al. (2009) determined a synergistic relationship between chronic inflammation and oxidative stress. Immune cells activated by proinflammatory factors produce a large number of ROS, which attack muscle fiber DNA, leading to mutation or deletion. At the same time, ROS further activates pro-inflammatory pathways, such as NF-κB, amplifies the inflammatory response, and ultimately weakens muscle fiber degeneration and fibrosis (Khansari et al., 2009).

Overall, the function of pro-inflammatory cytokines in muscle repair is not simply “beneficial or detrimental” but is determined by their expression timing and concentration. In the acute injury stage, TNF-α, IL-6, and other factors are essential “promoters,” which can induce repair by removing debris and activating satellite cells. However, if excessive use leads to cumulative damage, its maintained high expression will lead to chronic inflammation, destroy the repair microenvironment and inhibit the regeneration function. This mechanism suggests that clinical interventions should center on “temporal regulation”—avoiding the excessive suppression of pro-inflammatory factors during the acute phase while in the chronic phase seeking to downregulate their expression through targeted anti-inflammatory strategies or rehabilitation approaches (e.g., moderate exercise) to achieve a balance between “promoting repair and avoiding damage.”.

#### 3.3.2 Protein turnover: ubiquitin–proteasome system, autophagy, and mTOR signaling

The repair process of skeletal muscle after overuse injury is a dynamic balance between protein degradation (removal of damaged proteins and organelles) and synthesis required for structural rebuilding. Under physiological conditions, these two processes are coordinated and regulated through complex molecular pathways to maintain muscle mass and physiological stability. After injury, the degradation pathway preferentially removes necrotic proteins and then activates the synthesis pathway to reconstruct muscle fibers. If overuse disturbs this balance (degradation > synthesis), it can cause muscle atrophy, stalled repair, and even chronic dysfunction. The core modulation of this balance involves the interplay of the ubiquitin–proteasome system, autophagy pathways (degradation side), and mTOR signaling pathways (synthesis side), although much of the available mechanistic evidence is derived from acute injury, denervation, immobilization, or adjacent muscle disease models rather than chronic skeletal muscle OUI (Joshi et al., 2014).

After skeletal muscle injury, the ubiquitin–proteasome system (UPS) and autophagy are first activated to remove damaged proteins and organelles, creating space for subsequent synthesis and regeneration. Their activation mode is specific to the damage type. UPS is the key pathway for degradation of soluble weak proteins after injury. Abnormal proteins are eliminated by ubiquitin labeling and proteasome degradation. In an indirect rotator cuff injury model, Joshi et al. reported the activation of the ubiquitin–proteasome system, particularly when denervation was present; this evidence is relevant to muscle atrophy mechanisms but should not be interpreted as direct evidence for skeletal muscle OUI (Joshi et al., 2014). Autophagy, on the other hand, is vital for “bulk waste clearance” post-injury. The process includes forming autophagosomes that capture damaged mitochondria, myofibril pieces, and other elements, which subsequently merge with lysosomes for breakdown. Lundell et al. (2018), in a study on human spinal cord injury, observed a transient activation of the autophagy pathway (indicated by the LC3-I/LC3-II ratio) within the first month post-injury, aiding in the clearance of necrotic myofiber components. Nevertheless, after 3 months, autophagic activity normalized, but proteasome degradation stayed high. This sequence of “initial autophagy activation followed by ongoing proteasome activation” illustrates the timing of various degradation pathways (Lundell et al., 2018). It is critical to emphasize that degradation within a physiological scope is a necessary prerequisite for repair—insufficient degradation leads to the accumulation of impaired proteins and debris, impairing satellite cell migration and differentiation. Conversely, excessive degradation, like continuous UPS activation due to prolonged overuse, leads to a decrease in myofibrillar proteins, resulting in atrophy.

Following the clearance phase, the skeletal muscle begins protein synthesis by triggering the mTOR signaling pathway, which enhances myofiber rebuilding and satellite cell differentiation. The effectiveness of repair is directly influenced by the activity of this pathway. mTOR (especially mTORC1) acts as a “molecular switch” for anabolic pathways, activating translation–initiation pathways by phosphorylating downstream targets, such as 4E-BP1 and S6K1, thereby promoting the synthesis of contractile proteins, such as actin and myosin (Bond, 2016). According to Serova et al. (2024), triggering the mTOR pathway is crucial for improving muscle synthesis capacity. In C2C12 myotubes, BIO101 (an analoguex) enhanced the myotube diameter by 26% by activating the AKT/mTOR pathway and increased the core myogenic differentiation factors (MyoD, myogenin). Furthermore, it improved muscle contractility and motor skills in both adult and elderly mice, supporting the role of AKT/mTOR signaling in anabolic remodeling and myogenic differentiation (Serova et al., 2024). Chronic overuse can inhibit synthetic pathways through two processes: first, by lowering the levels of IGF-1 (insulin-like growth factor-1), which, in turn, decreases the upstream activation signals for AKT/mTOR, and second, by triggering AMPK (AMP-activated protein kinase), which competitively inhibits mTOR activity. Shenkman et al. (2019), in a study on alcoholic myopathy in women, reported that chronic alcohol exposure (modeling long-term metabolic stress and excessive injury) led to decreased IGF-1 levels, a 35%–45% decrease in the phosphorylation levels of AKT and 4E-BP1, and at the same time enhanced AMPK activity. This ultimately resulted in a reduction of the myofibers’ cross-sectional area, with atrophy affecting both fast-twitch and slow-twitch fibers, supporting the pathological sequence of “synthesis suppression → balance disruption → atrophy” (Shenkman et al., 2019).

The overall success of recovery after skeletal muscle overuse injury relies on the dynamic equilibrium between protein breakdown and production. In a physiological setting, repair follows the pattern of “initial degradation and removal, followed by synthesis and rebuilding.” Excessive use disrupts this equilibrium, leading to atrophy and dysfunction. Understanding the balance between UPS activity, autophagy, and mTOR signaling may help identify molecular targets for future rehabilitation-oriented interventions, although direct clinical validation in skeletal muscle OUI remains limited.

#### 3.3.3 Stress-responsive and myogenic transcriptional regulation

**Figure 6:**
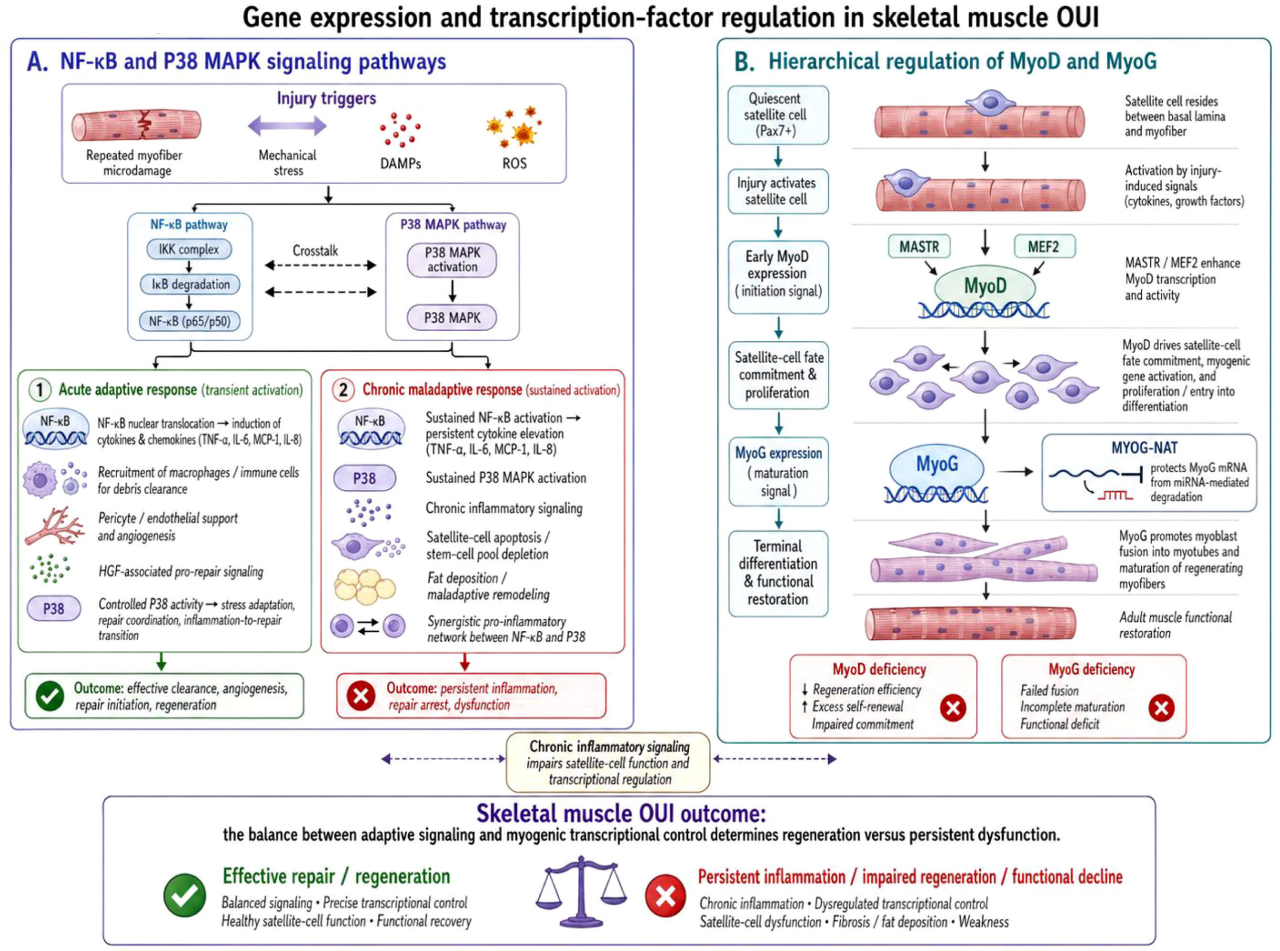
Myogenic transcription factors and satellite cell fate. **(A)** NF-κB and p38 MAPK signaling pathways in skeletal muscle OUI, showing the balance between adaptive signaling and chronic maladaptive activation. **(B)** Hierarchical regulation of MyoD and MyoG during satellite cell activation, myogenic commitment, differentiation, and myofiber maturation. Their activity is influenced by inflammatory, mechanical, and niche-related signals. Dysregulation of this transcriptional program may contribute to impaired regeneration, although direct evidence in chronic skeletal muscle OUI remains limited.

The regulation of gene expression and transcription factors is crucial in skeletal muscle overuse injuries. Transcriptional regulators coordinate inflammatory responses, satellite cell activation, myogenic commitment, differentiation, and remodeling after skeletal muscle injury. **Figure 6** summarizes myogenic transcriptional regulation and satellite cell fate in skeletal muscle OUI.

##### 3.3.3.1 NF-κB and p38 MAPK signaling

NF-κB and p38 MAPK are stress- and inflammation-responsive pathways that may participate in skeletal muscle injury, repair, and maladaptive remodeling (Tidball and Villalta, 2010; Proto et al., 2015; Vallecillo-Zúniga et al., 2021; Tu and Li, 2023). After myofiber damage, damage-associated molecular patterns, cytokines, oxidative stress, and mechanical stress can activate inflammatory and stress-response signaling, thereby promoting immune-cell recruitment, inflammatory mediator expression, and repair-related responses (Tidball and Villalta, 2010; Tu and Li, 2023). In acute injury models, time-limited activation of these pathways may contribute to debris clearance, angiogenic support, satellite cell activation, and coordination of early repair (Tidball and Villalta, 2010; LaBarbera et al., 2015; Peake et al., 2017; Tu and Li, 2023).

However, persistent or excessive activation of inflammatory and stress-response signaling may contribute to a maladaptive microenvironment. Sustained inflammatory signaling can promote catabolic activity, impair myogenic differentiation, increase oxidative stress, and reinforce fibrotic remodeling (Tidball and Villalta, 2010; Liu et al., 2017; Tu and Li, 2023). Evidence from dystrophic muscle and related chronic muscle disease models suggests that prolonged NF-κB activation may be associated with persistent inflammation and impaired muscle repair, but these models are not equivalent to chronic skeletal muscle OUI (Proto et al., 2015; Vallecillo-Zúniga et al., 2021).

The role of p38 MAPK in skeletal muscle OUI should also be interpreted cautiously. p38 MAPK is broadly involved in cellular stress responses, inflammatory signaling, apoptosis, and differentiation-related regulation. However, some available evidence comes from broad immune, inflammatory, or non-muscle cell models, which should be regarded only as general mechanistic context rather than direct evidence for skeletal muscle OUI (Clark et al., 2008; Chen et al., 2022). Therefore, NF-κB and p38 MAPK should be interpreted as plausible regulatory pathways rather than confirmed disease-specific drivers of chronic skeletal muscle OUI. Future studies should clarify their timing, cell-specific roles, and relationship to functional recovery under repeated loading and insufficient recovery conditions.

##### 3.3.3.2 MyoD, MyoG, and satellite cell fate

MyoD and MyoG, which are muscle-specific transcription factors, play crucial roles in the activation of satellite cells, differentiation into myogenic cells, and regeneration of myofibers after skeletal muscle overuse injury. They constitute a hierarchical regulatory axis of “upstream initiation-downstream maturation”: MyoD regulates the start of myogenesis and the determination of satellite cell fate, whereas MyoG facilitates myoblast fusion and the physiological maturation of myofibers, together ensuring the structural rebuilding and functional recovery of muscle tissue after injury (Zammit, 2017).

MyoD is a core member of the myogenic regulatory factor (MRFs) family. Its main function is to start the transition of satellite cells from a quiescent to a differentiating state by activating the expression of muscle-specific genes, inducing the myogenic differentiation mechanism. It acts as the “initial signal” for muscle repair post-injury. After an injury, MyoD expression shows precise timing and serves as an early indicator of satellite cell activation. Rantanen et al. (1995), using a rat gastrocnemius muscle contusion model, reported that MyoD-positive myoblasts were detec **Table 1** 2 h post-injury, while satellite cell proliferation (indicated by bromodeoxyuridine incorporation) began only after 24 h. The concept of “differentiation preceding proliferation” implies that initial MyoD expression triggers the differentiation process in inactive satellite cells without relying on cell division (Rantanen et al., 1995). Cooper et al. (1999) further reported that among activated satellite cells, approximately 60% at first express MyoD (some co-expressing Myf5). Following this, these cells either proceed through the cell cycle to generate additional myogenic progenitor cells or differentiate directly into myoblasts, indicating that MyoD functions as a “molecular switch” in satellite cell fate determination (Cooper et al., 1999). MyoD initiates transcription by attaching to promoter or enhancer areas of muscle-specific genes, such as actin and myosin light chain, steering cells towards a muscle cell phenotype. Megeney et al. (1996), in hybrid mouse models crossing MyoD-null mice with mdx (dystrophic) mice, reported that mice lacking both MyoD and dystrophin exhibited substantially impaired myopathy severity and premature death. Additional research indicated that the number of satellite cells in MyoD-null mice did not decline, but these cells were more inclined toward self-renewal rather than differentiation, leading to a reduction of over 50% in muscle regeneration efficiency after injury. This result supports the possibility that MyoD does not regulate satellite cell quantity but determines their “differentiation fate” (Megeney et al., 1996). Moreover, MyoD expression depends on regulation by upstream coactivators. According to Mokalled et al. (2012), MASTR (myocardin-associated transcription factor coactivator) can enhance the adult-muscle-specific enhancer of MyoD by binding to myocyte enhancer factor 2 (MEF2), thereby increasing its expression. Satellite-cell-specific deletion of MASTR led to downregulated MyoD expression, abnormal myoblast differentiation, excessive proliferation, and substantially hindered muscle regeneration capacity. The defect was further diminished by the concurrent reduction of MRTF-A, another factor in the myocardin family, indicating that MyoD function requires synergistic regulation from upstream coactivators (Mokalled et al., 2012).

Expressed after MyoD, MyoG is a target gene influenced by MyoD, essential for promoting myoblast fusion into myotubes and aiding in the maturation and functional recovery of muscle fibers. It is a core molecule facilitating the transition from “structural repair” to “functional restoration” in muscle tissue after injury (Zammit, 2017). The expression of MyoG is heavily reliant on the activation of MyoD and is primarily observed during the mid-to-late phases of myogenic differentiation, closely aligned with the process of myofiber maturation. According to Füchtbauer and Westphal’s 1992 research using a mouse muscle transplantation regeneration model, MyoG expression was initiated just before myoblasts fused into myotubes, and the protein stayed in the nuclei of regenerating myofibers. Immunostaining showed that MyoG-positive cells were all desmin-positive (a myoblast marker), but creatine kinase (a marker of myofiber maturation) was not yet detectable at that time—revealing that MyoG is a marker for the “initiation of the late differentiation phase,” preparing for myotube formation and physiological maturation (Füchtbauer and Westphal, 1992). Kami et al. (1995) further observed in a rat muscle injury model that MyoG mRNA was detectable as early as 6 h post-injury, preceding creatine kinase mRNA (which appeared after 12 h), and MyoG was expressed in both myoblasts and nascent myotubes, confirming its continuous role in the “differentiation–maturation” process (Kami et al., 1995). MyoG enhances the physiological refinement of myotubes by triggering the transcription of genes linked to myofiber maturation, such as troponin and myosin heavy chain. On the other hand, it sustains its maintained role during regeneration by modulating its own mRNA stability. Yin et al. (2023) found that the natural antisense transcript of MyoG (MYOG-NAT) safeguards the 3′UTR of MyoG mRNA from being degraded by competing with microRNAs like miR-128-2-5p, miR-19a-5p, and miR-19b-5p, thus maintaining MyoG production. Laboratory experiments demonstrated that increasing MYOG-NAT expression improved myoblast differentiation by 40%, whereas reducing MYOG-NAT in living organisms caused muscle fiber shrinkage and delayed regeneration. This outcome reveals a post-transcriptional regulatory mechanism for MyoG, enriching its physiological network (Yin et al., 2023). Moreover, in adult muscle regeneration, MyoG plays unique roles that are different from its functions in embryonic myogenesis. Meadows et al. (2008) reported that in mice with a postnatal MyoG knockout, muscle development appeared normal, yet the gene expression in muscle satellite cells was markedly altered, and the maturation rate of myofibers during regeneration was slower. Additional analysis revealed that in adult muscle, genes controlled by MyoG are more linked to the physiological upkeep of myofibers, such as energy metabolism and contractile efficiency, while in the embryonic phase, the focus is on differentiation. This indicates that MyoG’s role is specific to certain stages, emphasizing “functional repair” during adult muscle regeneration (Meadows et al., 2008).

MyoD and MyoG work together in a synergistic network where MyoD starts the downstream differentiation process by triggering MyoG transcription. MyoG, in turn, further improves the MyoD-induced differentiation process through feedback regulation. Zammit (2017) highlighted the importance of hierarchical regulation in muscle regeneration, noting that inadequate MyoD expression hinders satellite cell differentiation, leading to excessive proliferation. MyoG deficiency prevents myoblasts from fusing into mature myofibers, causing “structural incompleteness–functional deficit” (Zammit, 2017). The modulation of these transcription factors may represent a mechanistic research direction, but direct therapeutic evidence in chronic skeletal muscle OUI remains limited—for example, MASTR-related regulation of MyoD and MYOG-NAT-related regulation of MyoG provide mechanistic insight into myogenic regulation, but their therapeutic relevance to chronic skeletal muscle OUI remains speculative (Zammit, 2017; Yin et al., 2023). MYOG-NAT enhances MyoG mRNA stability by competing with miR-128-2-5p, miR-19a-5p, and miR-19b-5p for binding to its 3′UTR, promoting muscle development and regeneration. Hence, modulating these factors offers a theoretical basis for interventions in conditions such as muscular dystrophy and chronic OUIs (Zammit, 2017; Yin et al., 2023).

Overall, MyoD and MyoG are the “core regulatory duo” for myogenesis following skeletal muscle overuse injury: MyoD serves as the “starter,” deciding the differentiation path of satellite cells, whereas MyoG operates as the “implementer,” promoting myofiber development and physiological restoration. By working together hierarchically, they facilitate effective muscle tissue repair following injury. Gaining a thorough understanding of their regulatory mechanisms can provide key molecular targets for the targeted treatment of muscle injuries.

#### 3.3.4 Growth factor signaling and the satellite cell niche

Growth factors and stem cells are the “dual key drivers” of repair following skeletal muscle overuse injury: Growth factors act as “signaling molecules,” precisely modulating cell proliferation, migration, and differentiation to construct a microenvironment conducive to repair. Stem cells (especially muscle satellite cells) act as “functional cells,” sustaining stem cell pool stability via self-renewal while differentiating into myoblasts to rebuild impaired muscle tissue. The synergy between their “signal–cell” ensures efficient transition from structural repair to functional recovery of muscle tissue following injury (Dumont et al., 2015).

The FGF family is a key signaling molecule involved in the repair of skeletal muscle injuries. Its key functions are mediated by two main pathways: “regulating satellite cell fate” and “promoting angiogenesis,” offering the “cellular basis” and “nutritional support” for muscle regeneration, functioning as a critical bridge connecting injury signals to repair implementation (Farooq et al., 2021). FGF attaches to FGF receptors (FGFRs; e.g., FGFR1, FGFR4) on the satellite cell surface, activating downstream signaling pathways (e.g., MAPK/ERK, PI3K/AKT) to directly regulate the rhythm of satellite cell proliferation and differentiation. In the early stage of injury, FGF (especially FGF2) expression is swiftly increased. Its main role is to “maintain satellite cells in a proliferative state”—by suppressing the early expression of core myogenic differentiation factors (e.g., MyoG), it prolongs the satellite cell proliferation cycle, increases the number of myogenic progenitor cells, and reserves sufficient cellular resources for the following myofiber reconstruction (Motohashi and Asakura, 2014). During the repair process, the intensity of FGF signals gradually diminishes, and satellite cells shift from a “proliferative state” to a “differentiative state.” FGF can now enhance the differentiation of myogenic progenitors into myoblasts by increasing MyoD expression, which later merge into new myofibers. This regulatory pattern is especially important under pathological conditions—for instance, in Duchenne muscular dystrophy (DMD) models, exogenous FGF supplementation can partially reverse the decrease in satellite cell regenerative capacity—by enhancing satellite cell proliferative activity and decreasing stem cell pool depletion triggered by premature differentiation, thereby delaying the rate of myofiber degeneration (Shimizu-Motohashi and Asakura, 2014). Muscle regeneration requires a sufficient supply of oxygen and nutrients. FGF aids the healing process by encouraging blood vessel formation in the damaged region, providing microenvironmental support. FGF can directly promote the growth and movement of vascular endothelial cells, leading them to create tubular formations. Simultaneously, FGF can enhance the expression of vascular endothelial growth factor (VEGF), boosting the angiogenic effect via “FGF–VEGF synergy” (Messina et al., 2007). Although the evidence of Messina et al. (2007) focused on VEGF, it determined the close link between angiogenesis and muscle regeneration—an increase in capillary density in the injured area can enhance oxygen supply by 30%–40% and accelerate the delivery of nutrients (e.g., amino acids, glucose), offering the material basis for satellite cell proliferation/differentiation and muscle protein synthesis. Moreover, nascent blood vessels can aid the timely resolution of inflammation by transporting anti-inflammatory factors (e.g., IL-10), avoiding chronic inflammation from suppressing repair (Messina et al., 2007). As an upstream regulator of angiogenesis, FGF expression levels directly determine the effectiveness and extent of angiogenesis, thereby influencing the overall process of muscle regeneration.

Muscle satellite cells, which are dormant stem cells situated between the basement membrane and the plasma membrane of muscle fibers, are crucial for repairing skeletal muscle. They obtain muscle tissue regeneration and steady-state maintenance through the dynamic process of “quiescence activation proliferation differentiation self-renewal” (Kong et al., 2024). The self-renewal ability of satellite cells is the key to its long-term repair function. While meeting the needs of myoblasts in the process of damage repair, it also ensures that the stem cell bank is not exhausted (Dumont et al., 2015). Under physiological conditions, satellite cells are static and express Pax7 (a stem cell marker) but do not express differentiation-related factors. Following injury, damage-associated molecular patterns (DAMPs) and growth factors (e.g., FGF, IGF-1) act together to activate satellite cells: quiescent satellite cells swiftly enter the G1 phase, sustaining Pax7 expression while beginning to express MyoD (a marker of differentiation initiation), finishing the transition from “quiescent” to “activated” state (Kong et al., 2024). Activated satellite cells produce myogenic progenitor cells through symmetric or asymmetric division—symmetric division swiftly expands cell numbers, while asymmetric division produces one daughter cell that retains the stem cell characteristics (maintaining self-renewal) and one that is committed to differentiation (entering the myogenic program) (Kong et al., 2024). Myogenic progenitor cells express MyoG, cease proliferation, and fuse into myotubes, which eventually mature into nascent myofibers, replenishing the impaired area (Dumont et al., 2015; Kong et al., 2024). Motohashi and Asakura (2014) further reported that satellite cell differentiation displays “plasticity”—when repair demands are high (e.g., severe injury), myogenic progenitors may preferentially fuse into myofibers; in the later stages of repair, several cells re-enter quiescence, replenishing the stem cell pool to ensure repair capacity for following injuries (Motohashi and Asakura, 2014). Self-renewal is a core property distinguishing satellite cells from ordinary myoblasts, mainly mediated by “asymmetric division” and “niche microenvironment regulation.” During division, satellite cells utilize polarity-distributed fate determinants (e.g., Notch signaling components, Pax7) to confer different molecular characteristics to the two daughter cells—the daughter cell located near the muscle fiber basement membrane retains high Pax7 expression and active Notch signaling, sustaining quiescent stem cell properties; the daughter cell farther from the basement membrane displays decreased Pax7 expression and enhanced MyoD expression, entering the differentiation program (Dumont et al., 2015). The quiescence and self-renewal of satellite cells depend on their surrounding niche microenvironment (including muscle fibers, blood vessels, and extracellular matrix). CXCL12 secreted by vascular endothelial cells helps to maintain the quiescence of satellite cells. Fibronectin in the extracellular matrix regulates the balance between proliferation and self-renewal by binding to integrins on the surface of satellite cells (Motohashi and Asakura, 2014). In chronic OUI or myopathy (such as DMD), the self-renewal ability of satellite cells gradually decreases. On the one hand, repeated damage repair cycles lead to frequent satellite cell activation, increase the proportion of symmetrical division, and deplete the stem cell bank. On the other hand, the destruction of niche microenvironment (such as chronic inflammation and reduced vascularization) inhibits the quiescence and self-renewal of satellite cells, further promoting the functional decline (Shimizu-Motohashi and Asakura, 2014)—for instance, in DMD patients, the number of satellite cells increasingly decreases with disease progression, and the proliferation/differentiation capacity of the remaining cells decreases, causing continuous myofiber degeneration without positive repair.

Overall, growth factors and satellite cells form an important signaling–cellular network involved in skeletal muscle repair (Motohashi and Asakura, 2014; Dumont et al., 2015; Kong et al., 2024). Growth factors such as FGF, IGF-1, HGF, and VEGF may regulate satellite cell activity, angiogenesis, extracellular matrix remodeling, and the formation of a regenerative microenvironment (Messina et al., 2007; Motohashi and Asakura, 2014; Shimizu-Motohashi and Asakura, 2014; Liu et al., 2017; Farooq et al., 2021). Satellite cells provide the cellular basis for myofiber repair through activation, proliferation, differentiation, fusion, and self-renewal (Hill et al., 2003; Yablonka-Reuveni, 2011; Chang and Rudnicki, 2014; Motohashi and Asakura, 2014; Dumont et al., 2015; Kong et al., 2024). Dysregulation of this network may contribute to impaired regeneration during repeated loading and insufficient recovery, although direct evidence in chronic skeletal muscle OUI remains limited (Shimizu-Motohashi and Asakura, 2014; Dumont et al., 2015; Kong et al., 2024). Therefore, therapeutic strategies targeting growth factors or satellite cells should be regarded as experimental for chronic skeletal muscle OUI until validated in longitudinal human studies with functional outcomes (Hill et al., 2003; Yablonka-Reuveni, 2011; Chang and Rudnicki, 2014; Stilhano et al., 2015).

### 3.4 Therapeutic and rehabilitation strategies for skeletal muscle OUI

Therapeutic and rehabilitation strategies for skeletal muscle OUI should be interpreted according to clinical readiness, evidence strength, and relevance to skeletal muscle pathology. Current management remains centered on individualized load modification, graded rehabilitation, correction of biomechanical contributors, and progressive return to activity. Pharmacological and physical therapies may be used as adjunctive strategies for symptom control or rehabilitation support in selected cases, but their disease-modifying effects on chronic skeletal muscle OUI remain uncertain. Regenerative, gene-based, wearable-sensor-based, and AI-assisted approaches should be framed as emerging or experimental unless supported by direct clinical evidence in skeletal muscle OUI (Hill et al., 2003; Messina et al., 2007; Wong et al., 2007; Yablonka-Reuveni, 2011; Chang and Rudnicki, 2014; Lisowska et al., 2018; Morelli et al., 2018; Truppa et al., 2020; Uddin et al., 2021; Khan et al., 2024; Solaiman et al., 2024; Chen et al., 2025). From a rehabilitation perspective, load monitoring is important because injury risk depends not only on tissue pathology but also on the interaction among external load, tissue capacity, fatigue, movement control, and recovery (Bahr and Krosshaug, 2005; Halson, 2014; Drew and Finch, 2016). **Table 2** outlines rehabilitation and therapeutic strategies for skeletal muscle OUI according to clinical readiness and evidence limitations.

**Table 2:** Rehabilitation and therapeutic strategies for skeletal muscle OUI according to clinical readiness and evidence limitations.

Strategy	Suggested role	Clinical readiness	Evidence limitation	References
Load modification	Reduce repeated injurious stress, restore the load–recovery balance, and prevent recurrent aggravation	High	Requires individualized monitoring of symptoms, workload, fatigue, and recovery	(Kibler et al., 1992; Bahr and Krosshaug, 2005; Halson, 2014; Drew and Finch, 2016; Aicale et al., 2018)
Graded rehabilitation	Restore strength, tissue tolerance, movement control, fatigue resistance, and return-to-activity capacity	High	Protocols vary by muscle group, sport or occupation, injury severity, and recurrence risk	(Bahr and Krosshaug, 2005; Hägglund et al., 2005; Weerapong et al., 2005; Halson, 2014; Drew and Finch, 2016)
Correction of biomechanical contributors	Reduce uneven stress distribution, faulty movement patterns, and impaired coordination within the muscle–tendon–joint system	High to moderate	Requires individualized assessment; direct skeletal muscle OUI trials remain limited	(Taimela et al., 1990; Bahr and Krosshaug, 2005; Haggerty et al., 2014; Edwards, 2018; Earl-Boehm et al., 2020; Evangelidis et al., 2023)
Physical therapy modalities	Adjunctive symptom control and rehabilitation support	Moderate	Effects are parameter-dependent and often supported by heterogeneous or indirect evidence	(Nadler et al., 2004; Weerapong et al., 2005; Wong et al., 2007; Uddin et al., 2021)
NSAIDs and pharmacological analgesia	Short-term symptom relief in selected cases	Moderate	Timing, dose, duration, and tissue context may influence repair; disease-modifying effects in chronic skeletal muscle OUI are not established	(Garrick, 2011; Lisowska et al., 2018; Morelli et al., 2018; Solaiman et al., 2024)
Regenerative/biologic approaches	Potential repair support in severe or refractory injury	Low	Mostly preclinical, indirect, or extrapolative evidence; limited direct evidence in chronic skeletal muscle OUI	(Hill et al., 2003; Messina et al., 2007; Wong et al., 2007; Yablonka-Reuveni, 2011; Chang and Rudnicki, 2014; Motohashi and Asakura, 2014; Shimizu-Motohashi and Asakura, 2014; Dumont et al., 2015; Stilhano et al., 2015; Lisowska et al., 2018; Farooq et al., 2021; San Emeterio et al., 2021; Uddin et al., 2021; Kong et al., 2024; Solaiman et al., 2024; Wei et al., 2024)
Gene/RNA-based approaches	Experimental modulation of inflammatory, fibrotic, myogenic, or protein turnover pathways	Low	Safety, delivery efficiency, specificity, off-target effects, durability, and clinical indication remain unresolved	(Hill et al., 2003; Messina et al., 2007; Wong et al., 2007; Yablonka-Reuveni, 2011; Chang and Rudnicki, 2014; Motohashi and Asakura, 2014; Shimizu-Motohashi and Asakura, 2014; Stilhano et al., 2015; Lisowska et al., 2018; Farooq et al., 2021; Uddin et al., 2021; Solaiman et al., 2024)
Wearable sensors and AI-assisted feedback	Load monitoring, fatigue detection, movement feedback, and decision support	Emerging	Clinical outcome benefits in skeletal muscle OUI require validation	(Benson et al., 2020; Truppa et al., 2020; Rebelo et al., 2023; Khan et al., 2024; Chen et al., 2025; Di Paco and Cannataro, 2025; Rubio-Zarapuz et al., 2025)

Clinical readiness was categorized as follows: High = established clinical use and central to current management; Moderate = adjunctive use with mixed or context-dependent evidence; Low = limited direct evidence or mainly experimental application; Emerging = promising but not yet validated by clinical outcome studies in skeletal muscle OUI. “High to moderate” indicates established clinical relevance but limited direct trial evidence specific to skeletal muscle OUI.

#### 3.4.1 Load modification and graded rehabilitation

Load modification and graded rehabilitation are the most established components of skeletal muscle OUI management. Because skeletal muscle OUI develops when repeated loading exceeds adaptive and reparative capacity, early management should aim to reduce injurious load while avoiding unnecessary immobilization. Relative rest, temporary reduction of provoking activity, correction of faulty movement patterns, and gradual re-exposure to loading may help restore the balance between tissue stress and recovery (Kibler et al., 1992; Bahr and Krosshaug, 2005; Halson, 2014; Drew and Finch, 2016; Aicale et al., 2018).

Progressive rehabilitation should be individualized according to symptom severity, muscle group, functional demands, recurrence risk, and sport- or occupation-specific requirements. A staged approach is preferable: early rehabilitation should emphasize pain control, maintenance of safe range of motion, and avoidance of repeated aggravating load; the subacute phase should gradually reintroduce low-load strengthening, flexibility work, and neuromuscular control; and later stages should progress toward eccentric and concentric strengthening, endurance, coordination, and return-to-activity drills (Grgic et al., 2020). Progression should be based on functional tolerance rather than fixed timelines because recovery varies across individuals and injury contexts (Bahr and Krosshaug, 2005; Hägglund et al., 2005; Halson, 2014; Drew and Finch, 2016).

Return to activity should therefore be viewed as a criteria-based process rather than a time-based endpoint (Santos et al., 2023). Relevant criteria may include symptom response to loading, restoration of strength and endurance, movement quality, fatigue tolerance, and absence of recurrent pain during progressive task exposure. This approach is consistent with the broader load-management principle that injury risk reflects the interaction between external load, tissue capacity, fatigue, and recovery rather than load magnitude alone (Bahr and Krosshaug, 2005; Halson, 2014; Drew and Finch, 2016).

#### 3.4.2 Pharmacological and physical adjuncts

Pharmacological treatment should be considered an adjunctive option for symptom control in selected cases, not a cornerstone of skeletal muscle OUI management. NSAIDs may provide short-term pain relief after acute skeletal muscle injury, but their use in chronic skeletal muscle OUI should be cautious and time-limited. Evidence from acute skeletal muscle injury suggests that short-term NSAID use may reduce pain, but timing, dose, duration, and injury context are important because prolonged or inappropriate use may interfere with aspects of tissue repair (Messina et al., 2007; Morelli et al., 2018; Solaiman et al., 2024). Evidence from tendon-, ligament-, or bone-healing contexts should be interpreted as adjacent evidence and should not be directly generalized to skeletal muscle OUI (Messina et al., 2007; Lisowska et al., 2018).

Corticosteroids and biologic anti-inflammatory strategies should be discussed cautiously. Although they may reduce inflammation in selected musculoskeletal conditions, direct evidence supporting their routine use as disease-modifying treatments for chronic skeletal muscle OUI is insufficient. Because inflammation also participates in debris clearance and regeneration, excessive or poorly timed suppression of inflammatory signaling may theoretically impair repair. Treatment decisions should therefore balance short-term symptom relief against potential effects on tissue remodeling and regeneration (Messina et al., 2007; Tidball and Villalta, 2010; Peake et al., 2017; Tu and Li, 2023).

Physical therapy modalities may provide adjunctive support for pain control, tissue perfusion, mobility, and functional restoration, but they should not replace load modification and graded rehabilitation. Modalities such as therapeutic ultrasound, electrotherapy, heat/cold application, and laser-based approaches have been studied across heterogeneous musculoskeletal conditions, but the direct evidence base for chronic skeletal muscle OUI remains limited. Evidence from adjacent orthopedic models suggests that hyperbaric oxygen may influence tendon–bone interface healing, but such findings should be interpreted as indirect musculoskeletal evidence rather than as proof of efficacy for skeletal muscle OUI (Li et al., 2025). Therefore, these modalities should be presented as supportive tools whose effects depend on indication, parameters, timing, and integration into an active rehabilitation program (Wong et al., 2007; Uddin et al., 2021). Their use should be individualized and interpreted as symptom- or function-oriented rather than as proven disease-modifying therapy for skeletal muscle OUI.

#### 3.4.3 Regenerative, biologic, and gene-based approaches

Regenerative and biologic approaches may have theoretical relevance for severe or refractory muscle injury, particularly when impaired satellite cell function, inadequate regeneration, or fibrotic remodeling limits recovery. However, their application to chronic skeletal muscle OUI remains mostly preclinical or early translational. Satellite-cell-based and other stem-cell-oriented strategies provide important mechanistic insight into muscle repair, but challenges remain, including cell expansion, survival, engraftment, immune compatibility, niche integration, and functional restoration (Hill et al., 2003; Yablonka-Reuveni, 2011; Chang and Rudnicki, 2014; Dumont et al., 2015).

Growth factor and biologic strategies should also be interpreted cautiously. Factors such as IGF-1, FGF, HGF, and other myogenic regulators can influence satellite cell activation, proliferation, differentiation, angiogenesis, and extracellular matrix remodeling. Nevertheless, the timing, dose, delivery route, and tissue context are critical, and direct clinical evidence in chronic skeletal muscle OUI remains limited. These approaches should therefore be framed as mechanistically promising but not yet clinically established for skeletal muscle OUI (Hill et al., 2003; Messina et al., 2007; Yablonka-Reuveni, 2011; Chang and Rudnicki, 2014; Motohashi and Asakura, 2014; Shimizu-Motohashi and Asakura, 2014; Dumont et al., 2015; Liu et al., 2017; Kong et al., 2024).

Gene- and RNA-based interventions remain experimental in the context of skeletal muscle OUI. Approaches targeting inflammatory signaling, fibrosis, satellite cell fate, or protein turnover may provide useful proof-of-concept tools for understanding muscle repair, but their clinical translation faces major barriers, including safety, delivery efficiency, specificity, off-target effects, durability of response, and appropriate patient selection. At present, gene editing and RNA-based strategies should be discussed as future translational directions rather than clinically validated treatments for skeletal muscle OUI (Hill et al., 2003; Yablonka-Reuveni, 2011; Chang and Rudnicki, 2014; Stilhano et al., 2015).

#### 3.4.4 Wearable sensors and AI-assisted monitoring

Wearable sensors and AI-assisted monitoring may support load tracking, fatigue detection, movement feedback, and individualized rehabilitation adjustment. These tools may help clinicians identify excessive loading, poor movement control, delayed recovery, or abnormal fatigue responses, thereby supporting more precise load management. However, most evidence remains derived from technology validation studies, sports monitoring research, or adjacent rehabilitation settings rather than direct clinical outcome trials in skeletal muscle OUI (Benson et al., 2020; Truppa et al., 2020; Rebelo et al., 2023; Khan et al., 2024; Chen et al., 2025).

The main value of these technologies is monitoring and decision support rather than direct tissue repair. Wearable devices can quantify external load, movement patterns, electromyographic activity, pressure distribution, or fatigue-related changes, while AI-assisted systems may help integrate these data into individualized feedback and decision support (Di Paco and Cannataro, 2025; Rubio-Zarapuz et al., 2025). Nevertheless, device accuracy, signal validity, user adherence, data privacy, algorithm transparency, population generalizability, and clinical integration remain important challenges. Therefore, wearable sensors and AI-assisted systems should be presented as emerging tools that may improve rehabilitation monitoring, not as established disease-modifying interventions for skeletal muscle OUI (Benson et al., 2020; Truppa et al., 2020; Rebelo et al., 2023; Khan et al., 2024; Chen et al., 2025).

### 3.4.5 Integrated evidence-based rehabilitation framework

Overall, rehabilitation for skeletal muscle OUI should prioritize interventions with the strongest clinical readiness: load modification, graded rehabilitation, movement correction, and progressive return to activity. Pharmacological and physical modalities may be used as adjunctive strategies when clinically appropriate, but they should be interpreted cautiously because direct evidence for disease modification in chronic skeletal muscle OUI is limited. Regenerative, gene-based, wearable-sensor-based, and AI-assisted approaches are promising but remain emerging or experimental. A clinically useful framework should therefore match each intervention to its evidence strength, directness to skeletal muscle OUI, and stage of recovery.

This evidence-aware approach also helps avoid overgeneralization. Findings from acute eccentric injury, tendinopathy, bone or ligament healing, volumetric muscle loss, genetic myopathy, or general rehabilitation technology may inform mechanistic hypotheses, but they should not be treated as direct proof for chronic skeletal muscle OUI. Future intervention studies should combine longitudinal load monitoring, functional outcomes, imaging, molecular biomarkers, and standardized return-to-activity criteria to determine which strategies meaningfully improve skeletal muscle recovery and reduce recurrence risk.

### 3.5 Limitations and future directions

Several limitations should be recognized. First, direct human evidence for chronic skeletal muscle OUI remains limited, and many mechanistic insights are derived from acute eccentric exercise, traumatic muscle injury, denervation, immobilization, genetic myopathy, metabolic stress, or adjacent musculoskeletal disorders. Second, skeletal muscle OUI is likely heterogeneous across muscle groups, populations, loading patterns, and recovery conditions. Third, emerging interventions, including regenerative medicine, gene-based therapy, wearable sensors, and AI-assisted rehabilitation, remain preclinical or early translational unless supported by direct clinical outcome data. Future studies should combine longitudinal load monitoring, imaging, molecular biomarkers, functional outcomes, and standardized return-to-activity criteria to clarify which mechanisms are causal, modifiable, and clinically relevant.

## 4 Summary and outlook

### 4.1 Concluding remarks

Skeletal muscle OUI should be understood as a load-related failure of adaptation rather than as a simple extension of acute eccentric muscle damage or DOMS. Recurrent subthreshold loading, insufficient recovery, impaired satellite-cell-mediated repair, maladaptive inflammation, oxidative stress, mitochondrial dysfunction, and dysregulated ECM remodeling may interact over time to produce persistent pain, fatigue, weakness, reduced loading tolerance, and functional decline. Current clinical management should prioritize individualized load modification, graded rehabilitation, correction of biomechanical contributors, progressive strengthening, and criteria-based return to activity. Pharmacological and physical modalities may be useful adjuncts in selected cases, but their disease-modifying effects in chronic skeletal muscle OUI remain uncertain. Regenerative, gene-based, wearable-sensor-based, and AI-assisted approaches are promising but should be considered emerging or experimental unless supported by direct clinical outcome evidence. A key message of this review is that direct skeletal muscle evidence must be distinguished from indirect or extrapolative evidence derived from acute injury models, tendon or cartilage disorders, volumetric muscle loss, genetic myopathies, metabolic stress, toxic exposure, or cell studies. Future research should prioritize longitudinal human studies that integrate training-load monitoring, muscle function testing, imaging, molecular biomarkers, and standardized return-to-activity criteria.

### 4.2 Outlook

Future research on skeletal muscle OUI should move beyond broad descriptions of overuse injury and focus on mechanisms that are specific to chronic load-related skeletal muscle pathology. A priority is to clarify how recurrent subthreshold loading, insufficient recovery, satellite cell dysfunction, ECM remodeling, persistent low-grade inflammation, oxidative stress, mitochondrial impairment, and altered protein turnover interact over time. Longitudinal human studies are especially needed because many current mechanistic insights are derived from acute eccentric exercise, traumatic injury, volumetric muscle loss, burn injury, denervation, genetic myopathy, metabolic stress, or cell models rather than chronic skeletal muscle OUI itself. Multi-omics approaches, imaging, molecular biomarkers, wearable monitoring, and functional testing may help identify early signatures of failed adaptation and delayed repair. However, these approaches should be linked to clinically meaningful outcomes, including pain, fatigue tolerance, strength recovery, recurrence risk, return-to-activity progression, and long-term tissue function. Regenerative medicine, stem-cell-based approaches, gene editing, and RNA-based interventions may provide useful mechanistic tools or future therapeutic directions, but their safety, delivery, specificity, durability, and direct clinical relevance to skeletal muscle OUI remain unresolved. Future studies should therefore combine mechanistic precision with clinically grounded rehabilitation outcomes while clearly distinguishing direct skeletal muscle OUI evidence from indirect or extrapolative models.
